# Prevalence and Risk of Meningococcal Disease or Carriage During Mass Gatherings and Associated Travel: Systematic Review and Meta-Analysis

**DOI:** 10.3390/tropicalmed10080207

**Published:** 2025-07-25

**Authors:** Mohammed Samannodi, Hassan Alwafi, Jihad Muglan, Abdullah Tawakul, Rami M. Algahtani, Hani M. Almoallim, Ismail Ahmad Alghamdi, Majed Sameer Obaid, Amar Mohammad A. Alkhotani, Aous Sami Hayat Alhazmi, Heba M. Adly, Anas A. Khan, Fahad A. Alamri, Mohammed A. Garout

**Affiliations:** 1 Department of Medicine, Faculty of Medicine, Umm Al-Qura University, Makkah 24382, Saudi Arabia; mssamannodi@uqu.edu.sa (M.S.); jamuglan@uqu.edu.sa (J.M.); 2 Department of Pharmacology and Toxicology, College of Medicine, Umm Al-Qura University, Makkah 24382, Saudi Arabia; hhwafi@uqu.edu.sa; 3 Department of Internal Medicine, Faculty of Medicine, Umm Al-Qura University, Makkah 24382, Saudi Arabia; aatawakul@uqu.edu.sa (A.T.); rmgahtani@uqu.edu.sa (R.M.A.); 4 Department of Community Medicine and Pilgrims Health Care, College of Medicine, Umm Al-Qura University, Makkah 24382, Saudi Arabia; iaghamdi@uqu.edu.sa (I.A.A.); msobaid@uqu.edu.sa (M.S.O.); hmhasan@uqu.edu.sa (H.M.A.); 5 Medical Center, Umm Al-Qura University, Makkah 24382, Saudi Arabia; aousalhazmi@gmail.com; 6 Department of Emergency Medicine, College of Medicine, King Saud University, Riyadh 11421, Saudi Arabia; anaskhan@ksu.edu.sa; 7 Global Centre for Mass Gatherings Medicine, Ministry of Health, Riyadh 11176, Saudi Arabia; faabalamri@moh.gov.sa; 8 Department of Community Medicine, Faculty of Medicine, Umm Al-Qura University, Makkah 24382, Saudi Arabia; magarout@uqu.edu.sa

**Keywords:** meningococcus, mass gathering, hajj, travel, infection

## Abstract

Background: While efforts have been made to control meningococcal disease or carriage during mass gatherings (MGs), it is still a significant problem. This meta-analysis aims to assess the prevalence and predictors of meningitis carriage during MGs and travel. Methodology: PubMed, Scopus, Embase, and Cochrane were searched from their conception to January 2025. Cohort and cross-sectional studies assessing the prevalence of meningitis carriage and its serotype related to MGs and/or travel, and risk factors associated with its spread, were considered. The Newcastle–Ottawa scale was used for the quality assessment of studies. Results: Out of 1301 studies, 25 were considered for this meta-analysis. The largest geographic area involved was Saudi Arabia. A meta-analysis of 24 studies identified a pooled prevalence rate of meningococcal disease or carriage of 15.9% (95%CI: 4.45–27.4%) and the most frequent infecting organisms to be Serotype C (13.9%; 95%CI: −14.7 to 42.5; 4 studies) and A (11.5%; 95%CI: −2.13 to 25.2; 9 studies) among those at MGs or traveling. Age, gender, smoking history, and the vaccination status did not affect the infection risk. Conclusions: There is an increased prevalence of meningococcal disease and carriage, especially Serogroups A and C, associated with MGs and travel. New interventions and methodologies should be undertaken to control and prevent meningococcal disease or carriage transmission during such events.

## 1. Introduction

Meningococcal disease or carriage caused by *Neisseria meningitidis (N. meningitidis)* is a life-threatening condition that can lead to fatal complications such as meningitis, septicemia, and death [1]. It is a global health threat with varying risks by geographic region [2]. Meningococcal meningitis represented more than 400,000 cases globally and contributed to approximately 32,000 deaths in 2019 [3]. Its prevalence has been increasing with 422 cases of Serogroup Y reported in 2023 and 143 cases in the first quarter of 2024, indicating a significant upsurge, compared to the 81 cases reported during the same period in 2023 by the Centers for Disease Control and Prevention (CDC) in the United States of America [4].

Multiple factors, including mass gatherings (MGs), international travel, and migration, contribute to the spread of meningococcal infection. The World Health Organization (WHO) defines MGs as “an organized or unplanned event where the number of people attending is sufficient to strain the planning and response resources of the community, state or nation hosting the event” [5]. Similarly, the Center for Disease Control and Prevention (CDC) Yellow Book describes it as “large numbers of people (>1000) at a specific location, for a specific purpose” [6]. Practically speaking, an MG can be any assembly of people large enough to strain local resources [7], and we considered mass gathering (MG) events as the focus of interest. The increased movement of individuals, coupled with the close contact at large events, creates a unique environment conducive to the spread of meningococcal bacteria [8]. Recent studies and outbreaks have highlighted the significance of MGs, such as religious pilgrimages, festivals, and sporting events, as potential risk factors for meningococcal disease or carriage transmission [9]. Large crowds in confined spaces can facilitate the spread of respiratory droplets, which are the primary mode of transmission for *N. meningitidis* [10]. Reports of outbreaks during large public events, such as the Hajj pilgrimage, have brought global attention to the importance of vaccination and preventive measures when attending such events [8]. The risk is further compounded by travel to areas with a higher prevalence of meningococcal disease, particularly in the African meningitis belt [11].

Meningococcal disease or carriage remains a significant threat during MGs, particularly due to the increased risk of transmission in crowded settings. The Hajj pilgrimage is currently the only MG event where meningococcal vaccination is mandatory, with the Kingdom of Saudi Arabia requiring proof of quadrivalent (ACWY) vaccination for all pilgrims and workers [12]. Available meningococcal vaccines include conjugate (MenACWY), polysaccharide (MenACWY), and protein-based vaccines (MenB), each targeting different serogroups [13]. MenACWY vaccines protect against Serogroups A, C, W, and Y, while MenB vaccines are essential in regions where Serogroup B is prevalent [13]. Although other MGs like the Olympics or World Cup may issue health advisories, none currently enforce mandatory meningococcal vaccination policies similar to the Hajj pilgrimage [14].

Despite the growing recognition of these risks, the existing literature describing prevalence in relation to MGs and travel is scarce [14]. While several studies have investigated this issue [10,14,15], there is no comprehensive synthesis that quantifies the prevalence and risk factors linked to the spread of meningococcal disease or carriage during MGs or travel. Furthermore, while vaccination strategies have been implemented at several MGs, their effectiveness remains a subject of debate [16]. A systematic review and meta-analysis are necessary to consolidate findings from various studies, estimate the overall risk, and provide evidence-based recommendations for public health interventions.

This study aims to review the evidence on the prevalence and predictors of meningococcal disease or carriage and its serogroups during MGs and/or during travel. Additionally, we reviewed the evidence on the risk assessment from these studies.

## 2. Materials and Methods

This study was registered with PROSPERO (registration number CRD42025637006) [17]. We followed a Participant, Exposure, Comparator, Outcome, and Study (PECOS) design, as per the *Cochrane Handbook of Systematic Reviews* [18]. Findings were reported following the Preferred Reporting Items for Systematic Reviews and Meta-Analyses (PRISMA) statement 2020 [19].

### 2.1. Literature Search

A comprehensive search of PubMed/Medline (https://www.ncbi.nlm.nih.gov/pubmed/, accessed on 19 July 2025), Scopus (https://www.scopus.com/pages/home?display=basic#basic/, accessed on 19 July 2025), the Cochrane Library (http://www.cochranelibrary.com/, accessed on 19 July 2025), and Embase (https://www.embase.com/landing?status=yellow, accessed on 19 July 2025) from inception to 16 January 2025 was conducted. ProQuest Dissertations and Theses Global were also searched for any additional data. A bibliographic search of relevant previous review articles was performed to locate any relevant citations. The search strategy comprised all entry terms, Medical Subject Heading (MeSH) terms, and all keywords that belong to these two domains: (1) travel OR MG AND (2) meningococcus. A detailed search strategy is provided in Supplementary File S1. 

### 2.2. Eligibility Criteria

Observational studies, such as cohort (retrospective, prospective, or ambispective) and cross-sectional studies, were considered. Only studies published in the English language that assessed the prevalence/incidence of meningococcal disease or carriage related to MG event, and/or associated with travel, were evaluated. Adult studies and studies of adults involving only a minor pediatric proportion (<10%) were included in this review to ensure no studies involving adults were excluded. However, studies with only a pediatric population were excluded.

Exposure was defined as factors that could occur during an MG such as, but not limited to, close contact (person-to-person transmission, airborne, or droplet pathogens), poor ventilation, duration of exposure, and shared surfaces. The exposure of interest should be associated with an MG event. However, the condition, such as outbreaks, region-specific disease, and vaccination, were excluded. Weakened immunity was examined as a risk factor in the subjects reviewed.

The primary outcome measured was the prevalence of meningococcal disease or carriage and its serogroup. Studies that either reported the overall incidence of meningococcal disease/carriage or its serogroup or both were also included. However, we did not consider studies which reported the surveillance of outbreaks, pandemics, or endemics that were not related to an MG. We considered both the carriage of meningococcus and the confirmed cases of meningococcal disease or carriage as per the authors’ reports. Secondary outcomes were risk factors associated with meningococcal disease among those who attend an MG, mortality associated with the incidence of meningococcal disease, quality of life, and economic burden in the study population. Reviews, case studies, protocols, guidelines, and individual case data and their follow-up were excluded.

### 2.3. Study Selection and Data Extraction

Publications were retrieved from the aforementioned databases as RIS/CVS files and exported to Rayyan software [Cambridge, MA, USA] (https://rayyan.ai/) [20] for duplicate removal and title and abstract screening. Remaining publications were exported to an Excel file and screened for study eligibility. A predefined data extraction grid was used for data extraction. Extracted information included characteristics of the studies (author, year, country, study design, location of the study, and place of data collection), type of exposure, type of gathering, subject characteristics (age, gender, number of infected people, the total number of people at risk, vaccination status, mortality), type of samples obtained for diagnostic testing, number of people tested, infection status, and serotype. Two independent reviewers performed the study selection and data extraction. Any disagreement during the study selection process and data extraction was resolved through discussion with a third author. Duplicates were removed by matching publication titles and authors.

### 2.4. Risk of Bias

Two review authors independently assessed the methodological quality of all the included studies using the Modified Newcastle Ottawa scale for cross-sectional studies [21], adapted from Herzog R et al. [22]. The selection, comparability, and outcome domain had a maximum of 5 scores, 3 scores, and 2 scores, respectively, totaling to a maximum possible score of 10. A score of 8–10 was considered high-quality, 6–8 moderate, and <6 low-quality. Disagreements were resolved by discussion between the investigators involved or by a third reviewer.

### 2.5. Strategy for Data Synthesis

The qualitative data from the individual studies were summarized in narrative form in tables, and quantitative data was pooled by meta-analysis. Review Manager Software (RevMan, version 5.4; The Cochrane Collaboration, London, UK) [23] and STATA Version 16 (StataCorp LLC, College Station, TX, USA) [24] were used for conducting the meta-analysis. The outcome was collected in terms of the number of infected cases and total number of samples in order to calculate the prevalence rate. The outcome was reported as a pooled estimate with 95% confidence interval (CI). The risk estimates were collected and reported as risk ratio (RR) with 95% CI. The statistical heterogeneity of data was assessed using the I^2^ statistic and Cochrane *p* value. A random-effects model was used to perform the meta-analysis [18]. Subgroup analyses were performed based on the serotype, type of travel, vaccination status, study design, study duration/time, and the test sample. The meta-regression was based on the study design, study duration/time, and the test sample to explore the sources of heterogeneity. A sensitivity analysis was performed by excluding the studies with the smallest sample size (<25% of the total sample) [25,26] to check the robustness of the findings. A funnel plot was generated for visual inspection of publication bias for outcomes which had a minimum of 10 studies in the analysis. The Egger’s test was used to statistically confirm any publication bias [18].

## 3. Results

### 3.1. Study Selection Process

**Figure 1:**
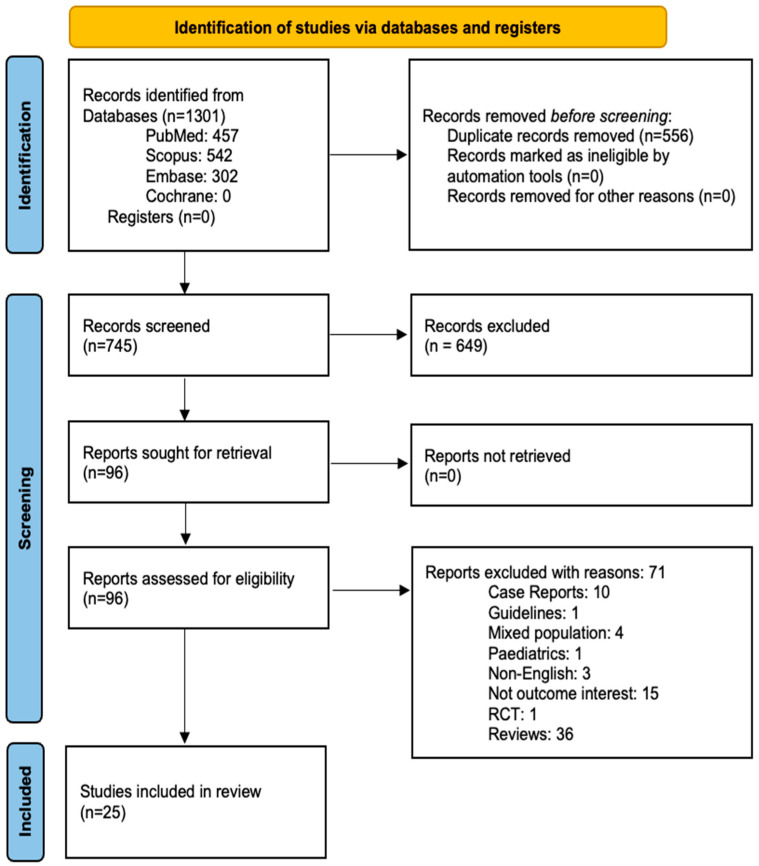
The PRISMA flow diagram showing the study selection process.

The literature search yielded a total of 1301 articles. After the removal of duplicates (n = 556) and the exclusion of non-relevant articles (n = 649) during the initial screening, 96 relevant articles were identified for full-text screening. Of these, 71 were excluded, as described in Supplementary File S1. The 25 remaining publications [27,28,29,30,31,32,33,34,35,36,37,38,39,40,41,42,43,44,45,46,47,48,49,50,51] met the study criteria. The PRISMA flow diagram of the study selection process is provided in Figure 1. The list of excluded studies is provided in Supplementary File S2. The study by Fazio et al. [32] was excluded from the overall prevalence analysis as their focus was the serogroup data, and the number of patients at risk was not clearly stated. However, this study was considered for the meta-analysis of serogroups.

### 3.2. Study Characteristics

A total of 25 studies were included in this systematic review, 9 of which were conducted in Saudi Arabia (n = 9; 36%). The included studies were prospective cohort (n = 14; 56%), retrospective cohort (n = 3; 12%), ambispective cohort (n = 1; 4%), and cross-sectional (n = 7; 28%) in nature. Gathering sites included airports (n = 8; 32%), home gatherings (n = 7; 28%), clinics/hospitals (n = 3; 12%), Muslim centers (n = 3; 12%), and a holy masjid (n = 1; 4%). The remaining three studies (12%) were collected from a public database. Most of the publications described subjects traveling and gathering for Hajj (n = 18; 72%), Umrah (n = 2; 8%), both Hajj and Umrah (n = 1; 4%), world travel (n = 3; 12%), or as refugee migration (n = 1; 4%). A detailed description of the characteristics is provided in Table 1.

### 3.3. Characteristics of the Included Participants

The number of people in each publication ranged from 15 to 38,849, with a total of 61,509 (median: 373, IQR: 216.5–801.5). Men and women were present in similar numbers. There were 109 (0.18%) study subjects <18 years of age across the entire included study populations in the analyses. All subjects reported were vaccinated in 14 (56%) of the 25 studies. The biological material tested was obtained by a throat swab (10 publications; 40%), a nasopharyngeal swab (3 publications; 12%), a oropharyngeal swab (7 publications; 28%), cerebrospinal fluid (CSF) (2 publications, 8%), blood and CSF (1 publication, 4%), and other clinical samples (2 publications; 8%) (Table 2). A total of 67,140 biological samples were collected where participants were either tested once or multiple times. Of these samples, 4895 (7.29%) were observed to be positive for meningococcus. A detailed description of the subjects and samples is provided in Table 1 and Table 2.

### 3.4. Methodological Quality of Included Studies

All the studies were graded to be high-quality, as per the modified Newcastle Ottawa scale for cross-sectional studies. The studies scored from 8 to 10, with an average score of 9.04. The major concerns were the confounding factors, as many studies did not report the age, gender, geographic location, time and duration of exposure, behavioral factors, previous health conditions, or vaccination history. No studies performed an independent blind assessment. A detailed methodological quality assessment is provided in Table 3.

### 3.5. Primary Outcomes

#### 3.5.1. Prevalence of Meningococcal Disease

**Figure 2:**
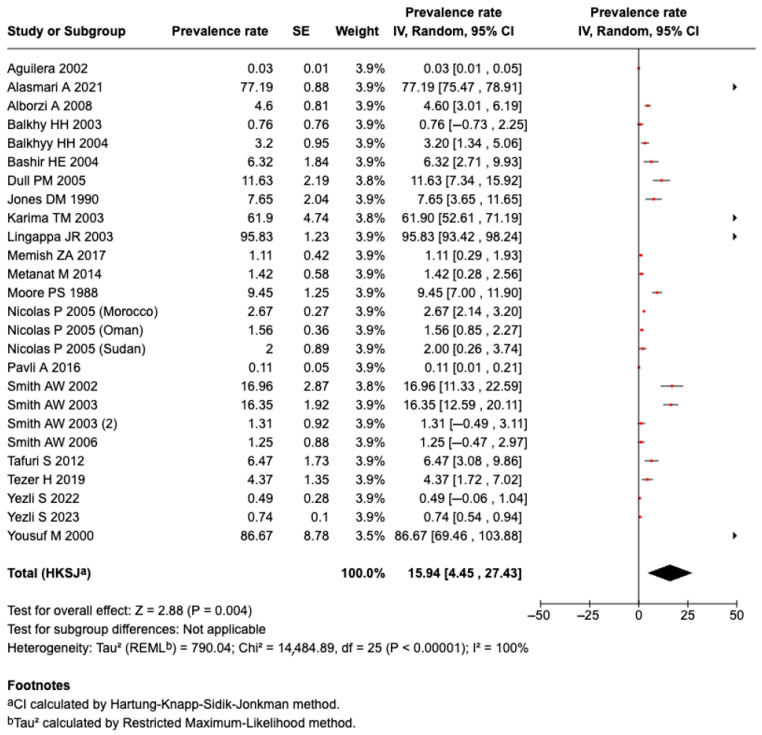
The meta-analysis of the prevalence of meningococcal disease or carriage [27,28,29,30,31,33,34,35,36,37,38,39,40,41,42,43,44,45,46,47,48,49,50,51].

A meta-analysis of 24 included studies demonstrated the pooled prevalence of meningococcal disease or carriage among those at risk as 15.9% (95%CI: 4.45–27.4%, I^2^: 100%), as shown in Figure 2. The subgroup analysis showed a higher prevalence of meningococcal disease or carriage among those at MGs (18.1%; 95%CI: 3.83–32.43; 19 studies) than among those that were traveling (4.41%; 95% CI: −5.92 to 14.7; 3 studies). Two publications reported people that traveled and attended MGs (9.99%; 95C% CI: 1.94 to 21.91). The publication heterogeneity was high for MGs (I^2^: 100%) and traveling (I^2^: 90%) separately; in contrast, the heterogeneity was 0% for studies where people traveled and attended MGs. The retrospective studies reported a higher prevalence of meningococcal disease/carriage than that of cross-sectional, ambispective, and prospective studies (53.75% vs. 14.04% vs. 6.32% vs. 3.57%). The studies conducted during 1990–2000 had a higher prevalence than that of studies conducted during 2000–2010 and 2010–2020 (29.58% vs. 14.49% vs. 11.43%). The results are presented in Supplementary Files S3–S5.

#### 3.5.2. Exploration of Heterogeneity Through Subgroup Analysis

The subgroup analysis based on the travel history, study design, study duration/time, and test sample type indicated that those analyses in which the studies that reported both travel and mass gatherings (I^2^: 0%), being an ambispective study (I^2^: 0%), the use of blood and CSF (I^2^: 0%) or a bacterial isolate and/or culture sample (I^2^: 0%) might have contributed to the heterogeneity. However, the studies that included participants during a mass gathering or travel only; were cross-sectional, prospective, and retrospective studies; were conducted between 1990 and 2000, 2000 and 2010, and 2010 and 2020; and used throat samples, oropharyngeal swabs, nasopharyngeal swabs (all with I^2^: >90%), and CSF (I^2^: 83.8%) might have not influenced the heterogeneity (Supplementary Files S3–S6).

#### 3.5.3. Results of Meta-Regression Analysis

The meta-regression indicated that the study duration/time, test sample, and study design did not significantly (*p* > 0.05) affect the heterogeneity in the analysis. The result of the meta-regression is provided in Table 4.

#### 3.5.4. Prevalence of Serogroup a Meningococcal Disease or Carriage

**Figure 3:**
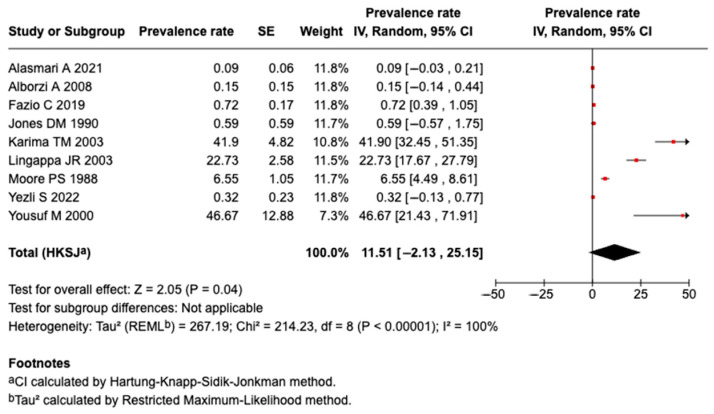
The meta-analysis of the prevalence of Serogroup A [27,31,32,35,43,44,49,50,51].

Nine studies reported Serogroup A meningococcal disease. A meta-analysis of the nine studies revealed that the pooled prevalence among those who traveled or attended an MG was 11.5% (95%CI: −2.13 to 25.15%, I^2^: 100%), as shown in Figure 3. There was a higher prevalence of Serogroup A meningococcal disease or carriage among those who attended an MG (13.1%; 95%CI: −2.43 to 28.65%, I^2^: 100%; n = 8 studies) than in those that traveled (0.59%; 95%CI: −0.57 to 1.75%; n = 1 study). However, there was no significant change in the heterogeneity, as shown in Supplementary File S7.

#### 3.5.5. Prevalence of Serogroup B Meningococcal Disease or Carriage

**Figure 4:**
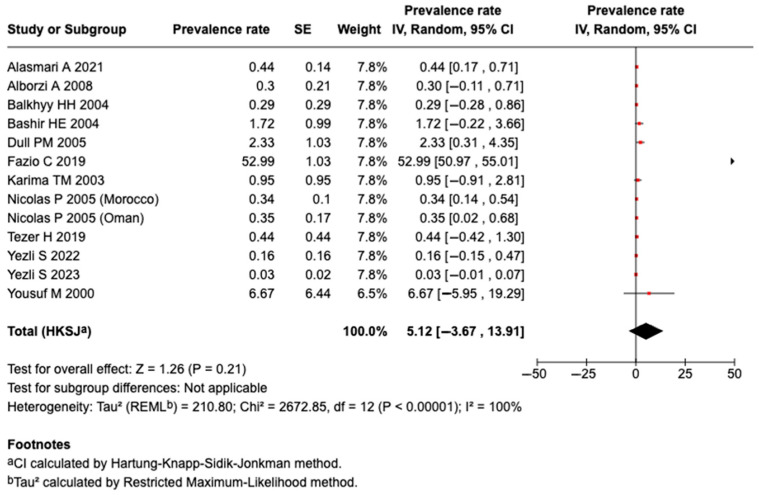
The meta-analysis of the prevalence of Serogroup B [27,29,30,31,32,35,36,39,40,43,46,51].

Twelve studies identified Serogroup B meningococcal disease. A meta-analysis of the 12 studies revealed that the pooled prevalence among those who traveled or attended an MG was 5.12% (95%CI: −3.67 to 13.91%, I^2^: 100%), as shown in Figure 4. There was a higher prevalence of Serogroup B meningococcal disease or carriage among those who attended an MG (5.36%; 95%CI: −4.28 to 15%, I^2^: 100%; n = 11 studies) than in those that traveled (2.33%; 95%CI: 0.31 to 4.35%; n = 1 study), as shown in Supplementary File S8.

#### 3.5.6. Prevalence of Serogroup W135 Meningococcal Disease or Carriage

**Figure 5:**
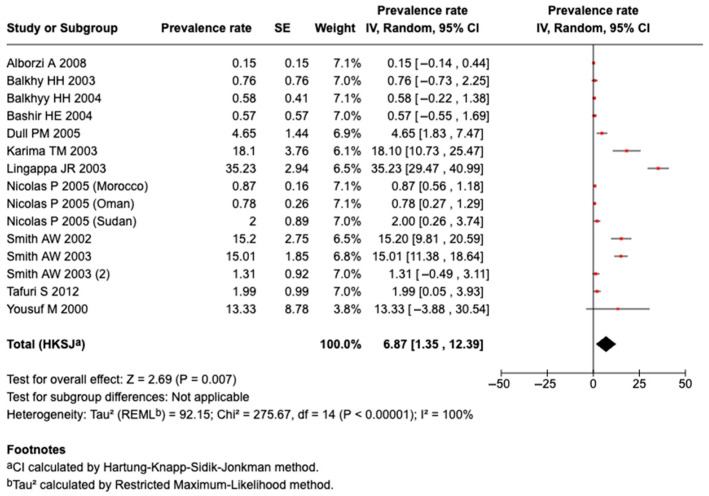
The meta-analysis of the prevalence of Serogroup W135 [27,28,29,37,39,40,41,43,44,45,46,47,51].

Twelve studies identified Serogroup W135 meningococcal disease. A meta-analysis of the 12 studies revealed that the pooled prevalence among those who traveled or attended an MG was 6.87% (95%CI: 1.35 to 12.39%, I^2^: 100%), as shown in Figure 5. There was a higher prevalence of Serogroup W135 meningococcal disease or carriage among those who attended an MG (7.51%; 95%CI: 1.05 to 13.96%, I^2^: 100%; n = 11 studies) than in those that traveled (1.99%; 95%CI: 0.05 to 3.93%; n = 1 study), as shown in Supplementary File S9.

#### 3.5.7. Prevalence of Serogroup C Meningococcal Disease or Carriage

**Figure 6:**
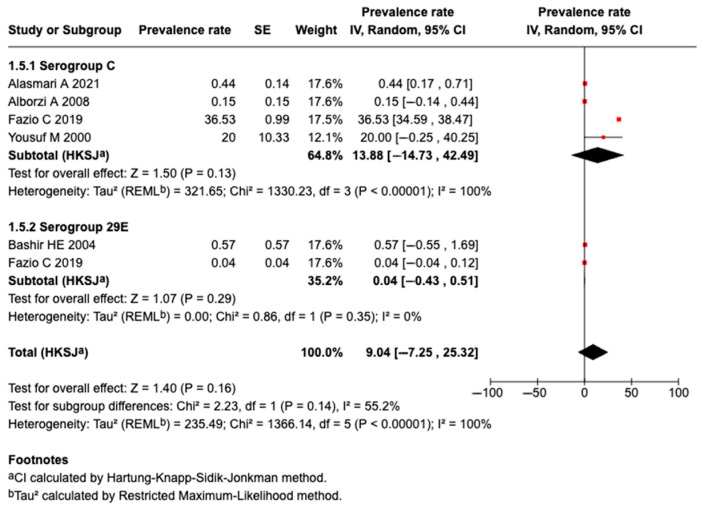
The meta-analysis of the prevalence of Serogroups C and 29E [27,31,32,40,51].

Four studies identified Serogroup C meningococcal disease or carriage. A meta-analysis of the four studies revealed that the pooled prevalence among those who traveled or attended an MG was 13.9% (95%CI: −14.73 to 42.49%, I^2^: 100%), as shown in Figure 6.

#### 3.5.8. Prevalence of Serogroup 29E Meningococcal Disease or Carriage

Two studies identified Serogroup 29E meningococcal disease or carriage. A meta-analysis of the two studies revealed that the pooled prevalence among those who traveled or attended an MG was 0.04% (95%CI: −7.25 to 25.32%, I^2^: 0%), as shown in Figure 6.

#### 3.5.9. Prevalence of Serogroup W Meningococcal Disease or Carriage

**Figure 7:**
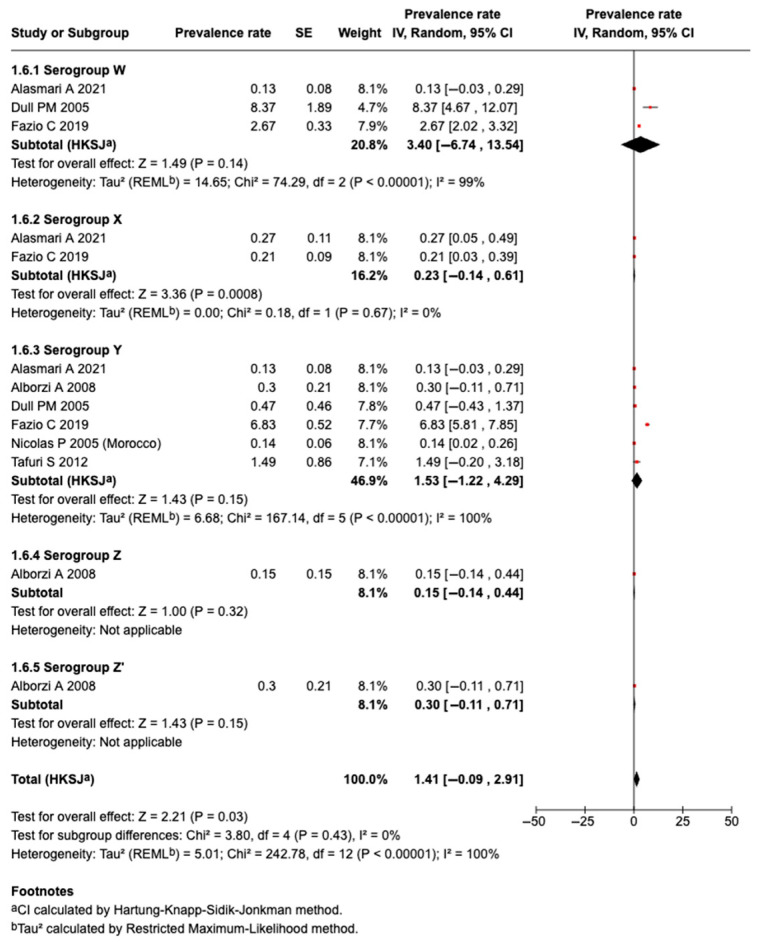
The meta-analysis of the prevalence of Serogroups W, X, Y, and Z [31,32,37,39,46,51].

Three studies identified Serogroup W meningococcal disease or carriage. A meta-analysis of three studies revealed that the pooled prevalence among those who traveled or attended an MG was 3.40% (95%CI: −6.74 to 13.54%, I^2^: 99%), as shown in Figure 7.

#### 3.5.10. Prevalence of Serogroup X Meningococcal Disease or Carriage

Two studies identified Serogroup X meningococcal disease or carriage. A meta-analysis of the two studies revealed that the pooled prevalence among those who traveled or attended an MG was 0.23% (95%CI: −0.14 to 0.61%, I^2^: 0%), as shown in Figure 7.

#### 3.5.11. Prevalence of Serogroup Y Meningococcal Disease or Carriage

Six studies identified Serogroup Y meningococcal disease or carriage. A meta-analysis of six studies revealed that the pooled prevalence among those who traveled or attended an MG was 1.53% (95%CI: −1.22 to 4.29%, I^2^: 100%), as shown in Figure 7.

#### 3.5.12. Prevalence of Serogroup Z Meningococcal Disease or Carriage

One study identified Serogroups Z and Z’ meningococcal disease or carriage. The prevalence rate of Serogroup Z was 0.15% (−0.14 to 0.44) and Serogroup Z’ was 0.30%, among subjects who attended an MG, as shown in Figure 7.

#### 3.5.13. Prevalence of Non-Groupable Meningococcal Disease or Carriage

**Figure 8:**
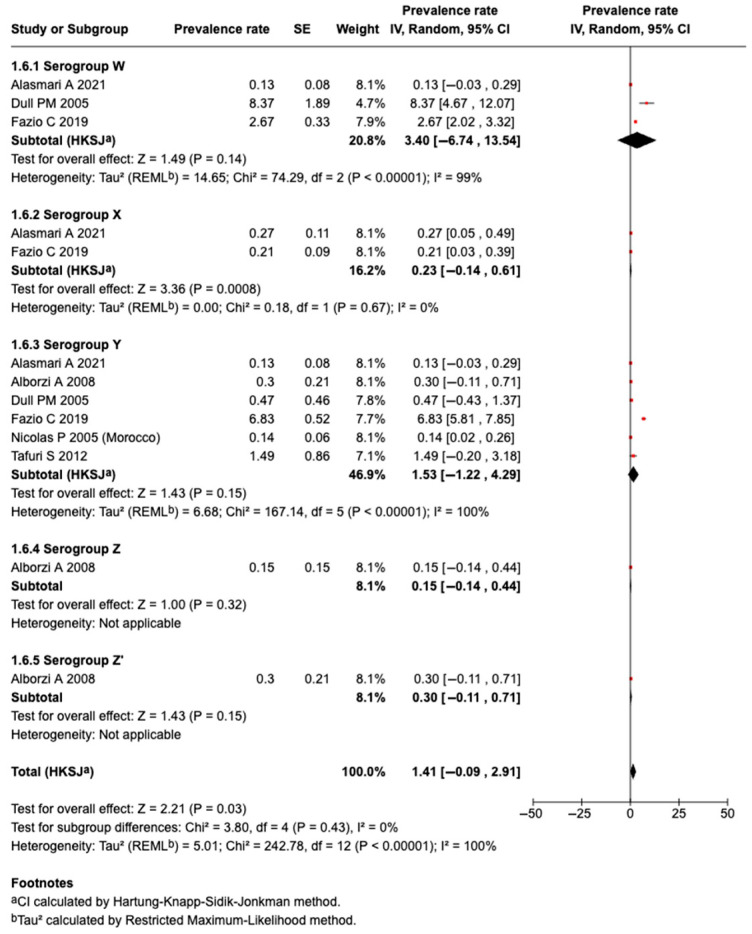
The meta-analysis of the prevalence of non-groupable and auto-agglutinable meningococcal disease or carriage [31,32,37,39,46,51].

Eleven studies identified non-groupable meningococcal disease or carriage. A meta-analysis of the 11 studies revealed that the pooled prevalence among those who traveled or attended an MG was 2.19% (95%CI: 0.91 to 3.47%, I^2^: 97%), as shown in Figure 8. There was a higher prevalence of non-groupable meningococcal disease or carriage among studies containing subjects who both traveled and attended an MG together (4.64%; 95%CI: −20.92 to 30.20%; I^2^: 79%; n = 2 studies) than in the nine studies reporting MGs alone (1.71%; 95%CI: 0.51 to 2.92%, I^2^: 96%; n = 9 studies). However, there was no significant change in the heterogeneity, as shown in Supplementary File S10.

#### 3.5.14. Prevalence of Auto-Agglutinable Meningococcal Disease or Carriage

Two studies identified auto-agglutinable meningococcal disease or carriage. A meta-analysis of the two studies revealed that the pooled prevalence among those who traveled or attended an MG was 1.59% (95%CI: −13.14 to 16.31%, I^2^: 67%), as shown in Figure 8.

#### 3.5.15. Effect of Vaccination on Prevalence of Meningococcal Diseases or Carriage

**Figure 9:**
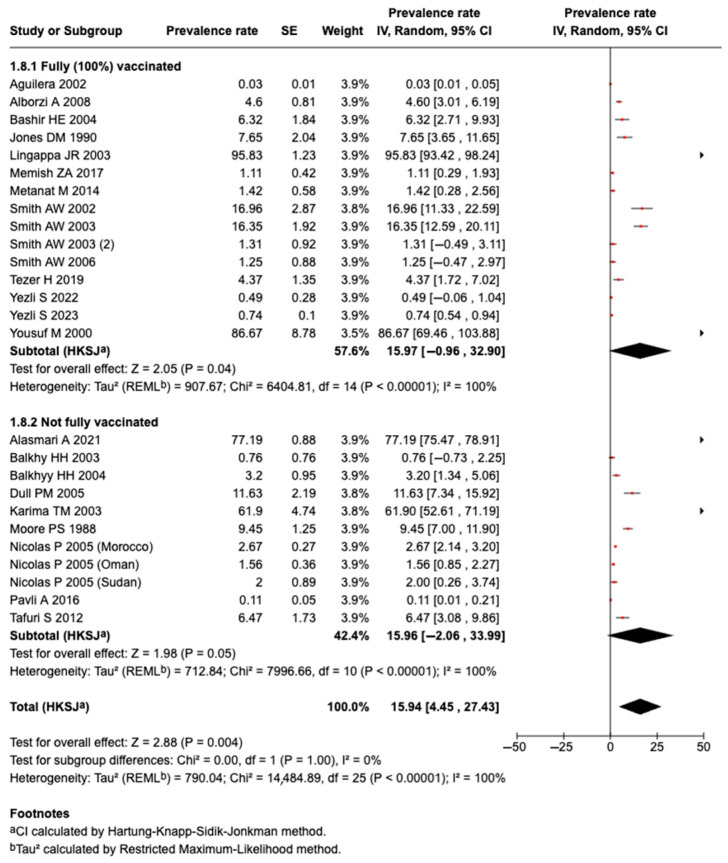
The effect of vaccination on the prevalence of meningococcal disease or carriage [27,28,29,30,31,33,34,35,36,37,38,39,40,41,42,43,44,45,46,47,48,49,50,51].

The prevalence of meningococcal disease or carriage among fully vaccinated people was 15.97% (95%CI: −0.96 to 32.90%; I^2^: 100% n = 15 studies) and was 16.0% (95%CI: −2.06 to 33.99%; I^2^: 100%; n = 9 studies) among those not vaccinated. However, there was no significant change in heterogeneity, as shown in Figure 9.

#### 3.5.16. Risk Factors Associated with the Prevalence of Meningococcal Disease or Carriage

Age, gender, smoking, and vaccination histories were evaluated as risk factors for meningococcal disease. None of them was identified as a risk factor (Age: 35–44 years of age [RR: 0.64; 95%CI: 0.16–2.56; I^2^: 0%; n = 2 studies; *p* = 0.692], 45–54 years of age [RR: 1.06; 95%CI: 0.37–3.10; I^2^: 0%; n = 2 studies; *p* = 0.661]; 55–64 years of age [RR: 0.98; 95%CI: 0.32–2.98; I^2^: 0%; n = 2 studies; *p* = 0.979], above 65 years of age [RR: 1.10; 95%CI: 0.24–5.11; n = 1 studies]; male gender [RR: 0.83; 95%CI: 0.43–1.58; I^2^: 34.99%; n = 3 studies; *p* = 0.215]; smoking history [RR: 1.10; 95%CI: 0.37–3.29; I^2^: 0%; n = 2 studies; *p* = 0.568]; and non-vaccinated status [RR: 1.48; 95%CI: 0.50–4.38; I^2^: 38.5%; n = 2 studies, *p* = 0.202]), as presented in Supplementary Files S9–S12.

### 3.6. Secondary Outcomes

#### 3.6.1. Mortality

Yousuf M et al. reported a mortality rate of 33%, with the poorest outcome in patients with a Serogroup W135 meningococcus infection [27]. Lingappa et al. reported a case fatality rate of 28% among all infected subjects (29% for Serogroup W-135 and 27% for Serogroup A) [44]. Jones DM et al. reported five deaths (13%) among 39 subjects diagnosed with Serogroup A infections [50]. Auguilera JF et al. reported the overall case fatality rate of European patients infected with Serogroup W-135 to be 15.6%, with a higher rate among subjects from the United Kingdom, 19%, and France, 16.7%, and a lower rate from other countries, 8.3% [48].

#### 3.6.2. Other Outcomes

No studies reported outcomes related to the quality of life or economic burden.

### 3.7. Publication Bias

The visual inspection of the funnel plots indicated a possible publication bias. This was statistically confirmed with Egger’s test (*p* < 0.05) for the outcome prevalence of meningococcal disease or carriage and its serogroup. The results are presented in Supplementary Files S13–S16.

### 3.8. Sensitivity Analysis

The sensitivity analysis was repeated after removing the two smallest studies. The prevalence of meningococcal disease or carriage in this subgroup was 13.9% (95%CI: 3.00–24.75%; I^2^: 100%; 22 studies). The exclusion of these two studies did not influence the study heterogeneity (Figure 2). The results of the sensitivity analysis are provided in Supplementary File S17.

## 4. Discussion

This systematic review and meta-analysis aimed to assess the prevalence and risk factors associated with meningococcal disease or carriage at MGs and during international travel. The meta-analysis of prevalence data was important because it supplied information about the current landscape of the disease and its associated contributors [25,26]. The findings from the reviewed studies underscore the elevated prevalence of meningococcal disease or carriage in people attending MGs and during travel. The risk of communicable disease transmission is high during MGs as there is close contact with multiple people from different geographical locations who can be infected, different immunity patterns, and different serotypes [52]. Shared accommodations, unhygienic practices, poor gathering etiquette, inadequate sanitation, a lack of cleanliness, smoking, and the overall health status of the participants have been described as risk factors for spread [53].

The Hajj and Umrah pilgrimages are among the largest MGs globally and have been linked to an increased risk of infectious diseases, including meningococcal infection [54]. The densely packed environment, the long duration of close contact, and international travel facilitate the transmission of Neisseria meningitidis during these events [55]. Multiple groups, including the World Health Organization, have recommended the use of pre-travel vaccination against meningococcal disease or carriage in order to prevent outbreaks during pilgrimages [56].

This analysis identified an overall pooled prevalence of meningococcal disease or carriage of 15.9%, with a higher proportion occurring among those attending MGs (18.1%). Serogroup C was most common (13.9%), followed by Serogroup A (11.5%). These findings are consistent with global surveillance data [57]. The higher prevalence of Serogroup C at mass gatherings aligns with previous reports linking this serogroup to large-scale outbreaks [58]. These results emphasize the importance of targeted vaccination strategies, particularly for Serogroups A and C, prior to attending MGs [59,60]. Meningococcal vaccination can reduce the risk of mortality and serious long-term sequelae [61], and efforts should be made to address the inadequate vaccinations [62].

In addition to the predominance of Serogroups A and C, the potential for Serogroup B (MenB) outbreaks at mass gatherings (MGs) such as the Hajj has been highlighted in the literature. Although historically less associated with large-scale outbreaks during Hajj, MenB remains a concern due to its unpredictable epidemiology and the lack of cross-protection from MenACWY vaccines. The literature has warned of the possibility of MenB emergence during Hajj, particularly as countries with rising MenB incidences contribute to the pilgrim population [63]. The availability of protein-based MenB vaccines (e.g., Bexsero and Trumenba) now offers targeted protection, especially in settings like universities and MGs where MenB transmission has been documented [64]. While these vaccines are not universally required for Hajj, their inclusion in pre-travel immunization strategies could mitigate the risk of MenB-related outbreaks.

The introduction of the MenAfriVac, a conjugate vaccine targeting Serogroup A, has significantly reduced the MenA incidence across the African meningitis belt, a region from which many Hajj pilgrims originate [65]. This success, combined with Saudi Arabia’s mandatory MenACWY vaccination policy for all Hajj pilgrims, has contributed to a marked decline in Serogroup A, C, W, and Y outbreaks during the pilgrimage [66]. The recent development of pentavalent MenABCWY vaccines, which offer a broader protection in a single formulation, presents a promising tool for future MG preparedness [67,68]. As MGs continue to grow in scale and diversity, integrating such vaccines into routine and event-specific immunization programs could enhance global health security and reduce the burden of meningococcal disease or carriage [14,69].

The prevalence of meningococcal disease or carriage during MGs, such as Hajj, varies by region. Some participants originate from countries with limited access to vaccines or do not adhere to vaccination recommendations [70]. MGs in countries with high endemicity, such as sub-Saharan Africa, are associated with a marked increase in disease risk [71]. Additionally, some of the analyses had a high prevalence rate of meningococcal infections. This might be due to the inclusion of surveillance studies which may not entirely represent the actual number of cases. This might have affected the overall outcome results.

Meningococcal vaccines, both conjugate and protein-based, have demonstrated a strong effectiveness in preventing invasive meningococcal disease, yet the longevity of the protection, particularly into adulthood, remains less clearly defined, especially when the primary immunization occurs in early adolescence [72]. National vaccination programs vary widely in terms of age groups targeted, included serogroups, and the overall uptake, creating gaps in protection that may leave some adults vulnerable during mass gatherings [73]. Given the heightened risk of meningococcal disease or carriage transmission in crowded settings, such as the Hajj pilgrimage and international festivals, tailored vaccination strategies including boosters and broader vaccine coverage should be considered for adults and older adults planning to attend these high-risk events [74].

Our evaluation of age, gender, smoking, and the vaccination status as risk factors for the spread of meningococcal disease or carriage did not identify any significantly associated risk. This finding is in contrast with previous research [75], which sometimes identified a younger age and a lack of vaccination as important determinants of risk. For example, younger individuals, particularly those under 25 years of age, have been found to be at a higher risk for meningococcal disease or carriage due to behaviors that increase close contact [76]. This finding needs to be interpreted with caution, as these results were obtained from prevalence studies which may not be an accurate design to ascertain a causal risk association. The lack of associated risk factors in our study may be due to the varied nature caused by including the geographical location of the events and differences in the race, country of origin, ethnicity, and hereditary and immune makeup of travelers. The high degree of the heterogeneity of these factors and the lack of detailed descriptions of all groups made the subgroup analysis difficult. Further study is needed to evaluate the role and interplay of these factors. There was little difference in the prevalence of meningococcal disease or carriage in fully vaccinated and partially vaccinated individuals we reviewed. These findings question the effectiveness of vaccination programs in preventing meningococcal disease or carriage during the MGs examined and travel. This finding may be explained by the inconsistent vaccine coverage among MG attendees, travel before an immune reaction has time to develop, or by the loss of vaccine efficacy over time, as the time from the last vaccination was not available for review [77]. Previous studies have shown that despite high vaccination rates, outbreaks still occurred due to factors such as an inadequate vaccine administration and the emergence of new serogroups [78,79]. Mortality was not assessed by many studies, which might be due to the cross-sectional nature of the studies. Further investigations are needed with long-term follow-up to better characterize our secondary outcome.

There are several limitations to this study. The evaluation of only English-language publications might have excluded some studies, especially regional-based studies. A comprehensive search strategy that used multiple databases was performed to minimize this risk. The quality of life and economic burden associated with meningococcal disease or carriage during MGs or travel were factors of interest to us; however, none of the studies reported this. It is important to note that the heterogeneity in this analysis was considerably high. However, this is expected due to the observational nature of the studies included and mixed populations. However, we conducted the subgroup analysis and sensitivity analysis to explore this high heterogeneity. The small amount of available data and the observational nature of studies (surveillance studies) used in the subgroup analyses and risk estimates might have affected our ability to detect a significant association. The detailed subgroup data was not available, as described above. Finally, this review is limited to meningococcal disease or carriage, IMD cases, and carriages at MG events that were sufficiently documented in the scientific literature, potentially underestimating the true burden. Events such as music and sporting festivals may be underrepresented, as illustrated by the briefly mentioned MenB case at the 2017 Boardmasters Festival in the UK [80].

The detailed vaccination data was not available in many studies. Other factors, such as the density of the crowded conditions, the duration of the exposure, the type of exposure during the MG, personal habits, and environmental factors should be explored in future studies. These findings suggest that further research is needed to identify other contributing factors and improve preventive strategies for meningococcal disease or carriage at mass-gatherings and during travel.

## 5. Conclusions

This study highlights the increased prevalence of meningococcal disease or carriage, especially Serogroups A and C, associated with MGs and travel. Age, gender, smoking, and vaccination status were not identified as risk factors for meningococcal disease or carriage in this study. The subgroup analyses and meta-regression did not rule out any factors that affect the heterogeneity. These findings underscore the need for the continued study of this problem and strategies to mitigate the risk of outbreaks during large-scale events and travel.

## Figures and Tables

**Table 1:** Characteristics of included studies.

Author, Year	Study Country	Study Design	Place of Data Collection	Type of Exposure	Type of Gathering	Country of Participants	Total Number of Participants	Male (%)	Age	Vaccinated (n)	Vaccination (%)
Balkhy HH, 2004 [29]	Saudi Arabia	Cross-sectional	Clinic/hospital	Mass Gathering	Hajj	Multi-national (n = 29)	344	45%	0–50 ***	245	71.2
Yezli S, 2023 [30]	Saudi Arabia	Prospective	Place of residence	Mass Gathering	Hajj	Multi-national (n = 13)	3921	52.4%	55 (13.4) *	3921	100.0
Alasmari A, 2021 [31]	Saudi Arabia	Cross-sectional	Airport	Mass Gathering	Hajj	Multi-national (n = 11)	2973	68%	11–65 ***	2117	71.2
Fazio C, 2019 [32]	Italy	Retrospective	Public database	Mass Gathering	Hajj	Italy	2357	NR	26 *	NR	N/A
Pavli A, 2016 [33]	Greece	Cross-sectional	Public database	Travel	World Travel	Greece	5283	64.30%	39.2 *	1150	21.8
Metanat M, 2015 [34]	Iran	Cross-sectional	Airport	Mass Gathering	Hajj	Iran	422	42.20%	21–95 ***	422	100.0
Yezli S, 2022 [35]	Saudi Arabia	Cross-sectional	Place of residence	Mass Gathering	Umrah	Multi-national (n = 17)	616	50.6%	19–91 *** (53.8) *	56	9.1
Tezer H, 2020 [36]	Turkey	Prospective	Airport	Mass Gathering	Hajj	Turkey	229	48.91%	23–71 *** 56 *	229	100.0
Tafuri S, 2012 [37]	Italy	Cross-sectional	Place of residence	Travel	Refugee Migration	Africa	253	88.50%	19.8 (6) *	NR	N/A
Memish ZA, 2017 [38]	Saudi Arabia	Prospective	Airport	Mass Gathering	Hajj	Multi-national (n = 10)	628	63.70%	18–65 ***	628	100.0
Dull PM, 2005 [39]	USA	Retrospective	Airport	Both	World Travel	USA	844	61.5%	Pilgrim: 47; non-pilgrim: 35 **	840	99.5
El-Bashir HE, 2004 [40]	UK	Ambispective	Muslim center	Mass Gathering	Hajj	UK	253	70%	42 (2–82) **	253	100.0
Wilder-Smith A, 2003 [41]	Singapore	Prospective	Muslim center	Mass Gathering	Hajj	Singapore	373	43%	47 (3–78) **	373	100.0
Wilder-Smith A, 2003 (2) [45]	Singapore	Prospective	Muslim Center	Mass Gathering	Hajj	Singapore	153	48%	48 (8.07) *	153	100.0
Wilder-Smith A, 2003 (3) [42]	Singapore	Prospective	Place of residence	Mass Gathering	Umrah	Singapore	160	36%	40 (2–80) **	160	100.0
Yousuf M, 2000 [27]	Saudi Arabia	Prospective	Holy Masjid	Mass Gathering	Hajj and Umrah	Multi-national (n = 7)	15	11%	NR	15	100.0
Karima TM, 2003 [43]	Saudi Arabia	Prospective	Clinic/hospital	Mass Gathering	Hajj	Multi-national (n = 26)	105	56.2%	20–80 ***	NR	N/A
Lingappa JR, 2003 [44]	Saudi Arabia	Retrospective	Public database	Mass Gathering	Hajj	Multi-national	264	53%	40 (0.2–80) **	264	100.0
Alborzi A, 2008 [51]	Iran	Prospective	Airport	Mass Gathering	Hajj	Iran	674	58.20%	52 *	674	100.0
Nicolas P, 2005 [46]	Morocco, Oman, and Sudan	Prospective	Place of residence	Mass Gathering	Hajj	Morocco	1186	NR	NR	NR	N/A
						Oman	399	NR	NR	NR	N/A
						Sudan	250	NR	NR	NR	N/A
Balkhy HH, 2003 [28]	Saudi Arabia	Prospective	Clinic/hospital	Mass Gathering	Hajj	Saudi Arabia	28	75%	18–61 ***	1	3.6
Wilder-Smith A, 2002 [47]	Singapore	Prospective	Place of residence	Mass Gathering	Hajj	Singapore	204	45%	48 (24–74) **	204	100.0
Aguilera, 2002 [48]	Europe	Prospective	Place of residence	Mass Gathering	Hajj	UK and France	38,849	NR	50	38849	100.0
Moore PS, 1988 [49]	USA	Cross-sectional	Airport	Both	World Travel	USA	550	Pilgrimage: 173	43.9 (19.1) *	Pilgrimage: 33/192	Pilgrimage: 17
Jones DM, 1990 [50]	UK	Prospective	Airport	Travel	Hajj	England and Wales	176	NR	NR	176	100

* Mean (SD), ** median (IQR), and *** range; NR: Not reported; N/A: Not applicable; UK: United Kingdom; and USA: United States of America.

**Table 2:** Meningococcal testing data with type of sample tested, number of samples tested, number of samples infected with meningococcus, and serogroup.

Author, Year	Study Duration	Test Sample	No. of People Tested (n)	Number Infected (n)	Serogroup (n)
Balkhy HH, 2004 [29]	2003	TS	344	11	**W-135:** 2; **B:** 1; **NG:** 8
Yezli S, 2023 [30]	2019	OPS	7842	58	**B:** 2; **NG:** 2
Alasmari A, 2021 [31]	2017	OPS	2249	130	**A:** 2; **B:** 10; **C:** 10; **W**: 3; **X**: 6; **Y:** 3
Fazio C, 2019 [32]	2000–2016	BIs and/or CS	2357	2357	**29E:** 1; **A:** 17; **B:** 1249; **C:** 861; **W**: 63; **X**: 5; **Y:** 161
Pavli A, 2016 [33]	2009–2013	TS	5283	6	NR
Metanat M, 2015 [34]	2012	OPS	422	6	NR
Yezli S, 2022 [35]	2019	OPS	616	3	**A:** 2; **B:** 1
Tezer H, 2020 [36]	2018	OPS	229	10	**B:** 1
Tafuri S, 2012 [37]	NR	NPS	201	13	**W-135:** 4; **Y:** 3; **Autoagglutinable:** 6
Memish ZA, 2017 [38]	2014	NPS	628	7	NR
Dull PM, 2005 [39]	2001	OPS	215	25	**W-135:** 10; **B:** 5; **W**: 18; **Y:** 1; **NG:** 15
El-Bashir HE, 2004 [40]	2000–2001	TS	174	11	**29E:** 1; **W-135: 1; B:** 3; **NG:** 6
Wilder-Smith A, 2003 [41]	2001	TS	373	61	**W-135:** 56
Wilder-Smith A, 2003 (2) [45]	2002	OPS	153	2	**W-135:** 2
Wilder-Smith A, 2003 (3) [42]	2001	TS	160	2	**Autoagglutinable:** 1; **NG:** 1
Yousuf M, 2000 [27]	1992–1993	CSF	15	13	**W-135:** 2; **A:** 7; **B:** 1; **C:** 3
Karima TM, 2003 [43]	2000	CSF	105	65	**W-135:** 19; **A:** 44; **B:** 1; **NG:** 1
Lingappa JR, 2003 [44]	2000	Blood and CSF	264	253	**W-135:** 93; **A:** 60
Alborzi A, 2008 [51]	2003	TS	674	31	**W-135:** 1; **A:** 1; **B:** 2; **C:** 1; **Y:** 2; **Z:** 1; **Z’:** 2; **NG:** 21
Nicolas P, 2005 [46]	2000–2001	TS	3558	95	**W-135:** 31; **B:** 12; **Y:** 5; **NG:** 47
Nicolas P. 2005 (Oman) *	2000–2001	TS	1157	18	**W-135:** 9; **B:** 4; **NG:** 5
Nicolas P. 2005 (Sudan) *	2000–2001	TS	250	5	**W-135:** 5
Balkhy HH, 2003 [28]	2001	TS	131	1	**W-135:** 1; **NG:** 5
Wilder-Smith A, 2002 [47]	2001	NPS	171	29	**W-135:** 26
Aguilera, 2002 [48]	2000	BI and/or CSs	38,849	12	NR
Moore PS, 1988 [49]	1987	TS	550	52	**A:** 36; **NG:** 16
Jones DM, 1990 [50]	1987	TS	170	13	**A:** 1; **NG:** 11

BI: Bacterial isolate; CSs: Clinical samples; CSF: Cerebrospinal fluid; NG: Non-groupable; NPS: Nasopharyngeal swab; NR: Not reported; OPS: Oropharyngeal swab; and TS: Throat swab. * The publication by Nicolas, P 2005 is presented showing the people originating from each of the 3 listed countries.

**Table 3:** Quality assessment of included studies.

Author, Year	Study Design	Selection (5 Score)	Comparability (2 Score)	Outcome (3 Score)	Total (10 Score)
Balkhy HH, 2004 [29]	Cross-sectional	5	1	3	9
Yezli S, 2023 [30]	Prospective	5	2	3	10
Alasmari A, 2021 [31]	Cross-sectional	5	1	2	8
Fazio C, 2019 [32]	Retrospective	5	1	3	9
Pavli A, 2016 [33]	Cross-sectional	5	2	2	9
Metanat M, 2015 [34]	Cross-sectional	5	2	3	10
Yezli S, 2022 [35]	Cross-sectional	5	2	3	10
Tezer H, 2020 [36]	Prospective	5	1	3	9
Tafuri S, 2012 [37]	Cross-sectional	5	1	2	8
Memish ZA, 2017 [38]	Prospective	5	2	3	10
Dull PM, 2005 [39]	Retrospective	5	2	2	9
El-Bashir HE, 2004 [40]	Ambispective	5	2	2	9
Wilder-Smith A, 2003 [41]	Prospective	5	2	3	10
Wilder-Smith A, 2003 (2) [45]	Prospective	4	2	3	9
Wilder-Smith A, 2003 (3) [42]	Prospective	5	2	3	10
Yousuf M, 2000 [27]	Prospective	5	1	3	9
Karima TM, 2003 [43]	Prospective	5	0	3	8
Lingappa JR, 2003 [44]	Retrospective	5	1	3	9
Alborzi A, 2008 [51]	Prospective	5	2	2	9
Nicolas P, 2005 [46]	Prospective	5	1	2	8
Nicolas P. 2005 (Oman) *	Prospective	5	1	3	9
Nicolas P. 2005 (Sudan) *	Prospective	5	1	3	9
Balkhy HH, 2003 [28]	Prospective	5	2	3	10
Wilder-Smith A, 2002 [47]	Cross-sectional	4	1	3	8
Aguilera, 2002 [48]	Prospective	5	0	3	8

* The publication by Nicolas, P 2005 is presented showing the people originating from each of the 3 listed countries.

**Table 4:** The results of the meta-regression.

Variable	Meta-Regression Coefficient	Standard Error	*p*-Value	95%CI
Study duration/time	−0.503	0.755	0.512	−2.071 to 1.063
Test sample	0.241	0.326	0.469	−0.436 to 0.917
Study design	0.332	0.741	0.658	1.205 to 1.870

## Data Availability

All the data and materials related to this article is available in this manuscript and Supplementary Files. Any additional data can be made available from the corresponding author on appropriate request.

## Supplementary Materials

The following supporting information can be downloaded at https://www.mdpi.com/article/10.3390/tropicalmed10080207/s1, Supplementary File S1: (A–D) Detailed search strategy; Supplementary File S2: List of excluded studies; Supplementary File S3: Prevalence of meningococcal disease or carriage based on travel history; Supplementary File S4: Prevalence of meningococcal disease or carriage based on study design; Supplementary File S5: Prevalence of meningococcal disease or carriage based on study duration/time; Supplementary File S6: Prevalence of meningococcal disease or carriage based on test sample; Supplementary File S7: Prevalence of serogroup A meningococcal disease or carriage based on travel history; Supplementary File S8: Prevalence of serogroup B meningococcal disease or carriage based on travel history; Supplementary File S9: Prevalence of serogroup W135 meningococcal disease or carriage based on travel history; Supplementary File S10: Prevalence of Non-groupable serogroup meningococcal disease or carriage based on travel history; Supplementary File S11: Risk of meningococcal disease or carriage based on age in years; Supplementary File S12: Risk of meningococcal disease or carriage for male gender; Supplementary File S13: Risk of meningococcal disease or carriage among those with any smoking history; Supplementary File S14: Risk of meningococcal disease or carriage among those with no vaccination; Supplementary File S15: Funnel plot for meningococcal disease or carriage; Supplementary File S16: Funnel plot for group B meningococcal disease or carriage; Supplementary File S17: Funnel plot for group W135 meningococcal disease or carriage; Supplementary File S18: Funnel plot for group W135 meningococcal disease or carriage; Supplementary File S19: Sensitivity analysis.

## Abbreviations

The following abbreviations are used in this manuscript:

CDC	Centers for Disease Control and Prevention
PROSPERO	International Prospective Register of Systematic Reviews
CRD	Part of the PROSPERO Registration Number
PECOS	Participant, Exposure, Comparator, Outcome, and Study
MeSH	Medical Subject Heading
IQR	Interquartile Range
CSF	Cerebrospinal Fluid
RR	Risk Ratio
RevMan	Review Manager Software
MGs	Mass Gatherings
