# Supplementary material for: Prevalence and Risk of Meningococcal Disease or Carriage During Mass Gatherings and Associated Travel: Systematic Review and Meta-Analysis
Source: Trop Med Infect Dis. 2025 Jul 25;10(8):207. doi: 10.3390/tropicalmed10080207 (PMC12389967; doi:10.3390/tropicalmed10080207)
Supplement: Supplementary file 1 [file tropicalmed-10-00207-s001.zip › tropicalmed-3681324-supplementary.pdf]

Supplementary Files

Supplementary File S1 A-D: Detailed search strategy

S1A: PubMed

Search number	Query	No. of hits
#1	"mass gathering*"OR "mass event*"[Title/Abstract] OR "crowd"[Title/Abstract] OR "hajj"[Title/Abstract] OR "Umrah"[Title/Abstract] OR "festival"[Title/Abstract] OR "concert"[Title/Abstract] OR "pilgrimage"[Title/Abstract] OR "social event"[Title/Abstract]	29,510
#2	"active travel"[Title/Abstract] OR "travelling"[Title/Abstract] OR "cycling"[Title/Abstract] OR "bicycling"[Title/Abstract] OR "walking"[Title/Abstract] OR "travel*"[Title/Abstract]	275,342
#3	#1 OR #2	304,023
#4	"meningococcus*" OR "meningococcal*" OR "Meningococcal Infection" OR "Meningococcal Disease" OR "meningococc*"	18,396
#5	#3 AND #4	457

S1B: Scopus

21	((TITLE-ABS-KEY ("mass gathering*" OR "mass event*" OR "crowd" OR "hajj" OR "umrah" OR "festival" OR "concert" OR "pilgrimage" OR "social event")) OR (TITLE-ABS-KEY ("active travel" OR "travelling" OR "cycling" OR "bicycling" OR "walking" OR "travel*"))) AND (TITLE-ABS-KEY ("meningococcus*" OR "meningococcal*" OR "meningococcal infection" OR "meningococcal disease" OR "meningococc*")) AND (LIMIT-TO (DOCTYPE , "ar")) Show less	542 results	Set alert More
20	((TITLE-ABS-KEY ("mass gathering*" OR "mass event*" OR "crowd" OR "hajj" OR "umrah" OR "festival" OR "concert" OR "pilgrimage" OR "social event")) OR (TITLE-ABS-KEY ("active travel" OR "travelling" OR "cycling" OR "bicycling" OR "walking" OR "travel*"))) AND (TITLE-ABS-KEY ("meningococcus*" OR "meningococcal*" OR "meningococcal infection" OR "meningococcal disease" OR "meningococc*")) Show less	1,057 results	Set alert More
19	((TITLE-ABS-KEY ("mass gathering*" OR "mass event*" OR "crowd" OR "hajj" OR "umrah" OR "festival" OR "concert" OR "pilgrimage" OR "social event")) OR (TITLE-ABS-KEY ("active travel" OR "travelling" OR "cycling" OR "bicycling" OR "walking" OR "travel*"))) AND (TITLE-ABS-KEY ("meningococcus*" OR "meningococcal*" OR "meningococcal infection" OR "meningococcal disease" OR "meningococc*")) Show less	1,057 results	Set alert More
18	TITLE-ABS-KEY ("meningococcus*" OR "meningococcal*" OR "meningococcal infection" OR "meningococcal disease" OR "meningococc*") Show less	25,025 results	Set alert More
17	(TITLE-ABS-KEY ("mass gathering*" OR "mass event*" OR "crowd" OR "hajj" OR "umrah" OR "festival" OR "concert" OR "pilgrimage" OR "social event")) OR (TITLE-ABS-KEY ("active travel" OR "travelling" OR "cycling" OR "bicycling" OR "walking" OR "travel*")) Show less	1,161,374 results	Set alert More
16	TITLE-ABS-KEY ("active travel" OR "travelling" OR "cycling" OR "bicycling" OR "walking" OR "travel*")	1,038,250 results	Set alert More
11	TITLE-ABS-KEY ("mass gathering*" OR "mass event*" OR "crowd" OR "hajj" OR "umrah" OR "festival" OR "concert" OR "pilgrimage" OR "social event") Show less	129,722 results	Set alert More

S1C: Embase

Number	Query	No. of hits
#1	'mass gathering'/exp OR 'mass gathering'	1,258
#2	'travel'/exp OR 'travel'	110,185
#3	#1 OR #2	111,192
#4	'meningococcosis'/exp OR 'meningococcosis' OR 'meningococcal disease'	16,413
#5	#3 AND #4	638
#6	#5 AND ('article'/it OR 'article in press'/it OR 'short survey'/it	302

S1D: Cochrane

Number	Query	No. of hits
#1	MeSH descriptor: [Mass gathering] explode all trees	3
#2	MeSH descriptor: [Meningococcal infection] explode all trees	567
#3	#1 AND #2	0

Supplementary File S2: List of excluded studies

No.	Title	Reason for exclusion
1	Epidemic meningococcal disease and travel	Review
2	Hajj-associated infections	Review
3	Meningococcal disease in travelers: a rare but devastating disease	Review
4	Prevention of meningococcal disease at mass gatherings: Lessons from the Hajj and Umrah	Review
5	Incidence and Prevention of Invasive Meningococcal Disease in Global Mass Gathering Events	Review
6	The risk of meningococcal disease in travelers and current recommendations for prevention	Guideline/Recommendation
7	Risk of antibiotic resistant meningococcal infections in Hajj pilgrims	Review
8	Emergence of invasive meningococcal disease during Hajj pilgrimage - vigilance and preparedness, in the post-pandemic year	Review
9	Invasive meningococcal disease and travel	Review
10	W135 meningococcal carriage in Hajj pilgrims	Review
11	Addressing the risk of global spread of Neisseria Meningitidis: strategies for the forthcoming 2024¬†Hajj following cases in the UK, France, and the USA	Review
12	Meningococcal disease and prevention at the Hajj	Review
13	Serogroup W-135 meningococcal disease among travelers returning from Saudi Arabia--United States, 2000	Review
14	Risk of transmitting meningococcal infection by transient contact on aircraft and other transport	Review
15	Meningococcal disease during the Hajj and Umrah mass gatherings: A, C, W, Y may be covered but don't forget the B and X factors!	Review
16	Travel-related Neisseria Meningitidis serogroup W135 infection, France	Review
17	Meningococcal disease in international travel: vaccine strategies	Review
18	Epidemiology of meningococcal disease in light of recent Hajj-associated outbreaks	Review
19	Outbreaks of meningococcal meningitis during Hajj: changing face of an old enemy	Review
20	[Meningococcal W135 infection epidemics associated with pilgrimage to Mecca in 2000]	Not English
21	[Meningococcal arthritis (Serogroup A) contracted during a pilgrimage to Mecca]	Not English
22	Cases of Meningococcal Disease Associated with Travel to Saudi Arabia for Umrah Pilgrimage - United States, United Kingdom, and France, 2024	Mixed age Population
23	Disease transmission and mass gatherings: a case study on	Not outcome of

	meningococcal infection during Hajj	interest
24	Neisseria Meningitis serogroup W135 in a traveler visiting Japan from Argentina, 2019	Review
25	Meningococcal disease outbreak related to the World Scout Jamboree in Japan, 2015	Not outcome of interest
26	Notes from the Field: Meningococcal Disease in an International Traveler on Eculizumab Therapy - United States, 2015	Case Report
27	Meningococcal disease during the Hajj and Umrah mass gatherings	Review
28	Prevention of meningococcal disease during the Hajj and Umrah mass gatherings: Past and current measures and future prospects	Review
29	A cohort study of the impact and acquisition of naspharyngeal carriage of Streptococcus pneumoniae during the Hajj	Not outcome of interest
30	Meningococcal and pneumococcal carriage in Hajj pilgrims: Findings of a randomized controlled trial	RCT
31	Meningococcal vaccine for hajj pilgrims: Compliance, predictors, and barriers	Not outcome of interest
32	Patterns of communicable and non-communicable diseases in pilgrims during Hajj	Review
33	Meningococcemia due to the 2000 Hajj-associated outbreak strain (serogroup W-135 ST-11) with immunoreactive complications	Review
34	Outbreak of Neisseria Meningitis capsular group W among scouts returning from the World Scout Jamboree, Japan, 2015	Pediatric population
35	Meningococcal serogroup A, C, W, and Y serum bactericidal antibody profiles in Hajj pilgrims	Not outcome of interest
36	Serogroup B meningococcal disease during Hajj: Preparing for the worst scenario	Review
37	Invasive meningococcal disease with fatal outcome in a Swiss student visiting Berlin.	Case Report
38	Characterization of serogroup A Neisseria Meningitidis from invasive meningococcal disease cases in Canada between 1979 and 2006: Epidemiological links to returning travelers	Not outcome of interest
39	French Hajj pilgrims' experience with pneumococcal infection and vaccination: A knowledge, attitudes and practice (KAP) evaluation	Not outcome of interest
40	A prolonged outbreak of invasive meningococcal disease in an extended Irish Traveler family across three Health Service Executive (HSE) areas in Ireland, 2010 to 2013	Not outcome of interest
41	Carriage of Neisseria meningitidis in the Hajj and Umrah mass gatherings	Review
42	Prevalence of MERS-CoV nasal carriage and compliance with the Saudi health recommendations among pilgrims attending the 2013 Hajj	Not outcome of interest
43	Meningococcal disease--probable transmission during an international flight.	Review
44	Surveillance for meningococcal carriage by Muslims returning from the Hajj to Hat Yai Airport, Thailand	Review

45	An estimation of imported infections concerning 2002 FIFA world cup Korea/Japan	Not outcome of interest
46	A case of sepsis by Neisseria meningitidis beginning with pneumonia during a trip abroad	Case Report
47	Comparison of serogroup W-135 meningococci isolated in Sweden during a 23-year period and those associated with a recent hajj pilgrimage	Not outcome of interest
48	Exposure to patients with meningococcal disease on aircrafts--United States, 1999-2001.	Case Report
49	Risk for meningococcal disease associated with the Hajj 2001.	Review
50	Meningococcal disease associated with the Haj--update.	Review
51	Meningococcal infection in pilgrims returning from the Haj: update.	Review
52	Serogroup W135 meningococcal disease in Hajj pilgrims	Review
53	Fatal meningococcaemia due to group W135 amongst Haj pilgrims: Implications for future vaccination policy	Case Report
54	Neisseria meningitidis serogroup W-135 isolated from healthy carriers and patients in Sudan after the Hajj in 2000	Not outcome of interest
55	W135 meningococcal carriage in association with the Hajj pilgrimage 2001: The Singapore experience	Review
56	Hajj-associated outbreak strain of Neisseria meningitidis serogroup W135: Estimates of the attack rate in a defined population and the risk of invasive disease developing in carriers	Not outcome of interest
57	Bacterial meningitis exposure during an international flight: Lessons for communicable pathogens	Review
58	Hajj-related Neisseria meningitidis serogroup W135 in Mauritius	Case Report
59	Meningococcal infection in children visiting Majorca.	Pediatrics
60	From the Centers for Disease Control and Prevention. Risk for meningococcal disease associated with the Hajj 2001.	Review
61	Neisseria meningitidis serogroup W135 epidemic related to the 2000 Haj, France	Case Report
62	From the Centers for Disease Control and Prevention. Update: Assessment of risk for meningococcal disease associated with the Hajj 2001.	Case Report
63	W135 meningococcal disease in England and Wales associated with Hajj 2000 and 2001	Not outcome of interest
64	Evaluation of travelers returning from the 1992 olympics in barcelona, spain: Did they acquire resistant pneumococci and meningococci?	Not outcome of interest
65	Determinants of case fatality rates of meningococcal disease during outbreaks in Makkah, Saudi Arabia, 1987-97	Mixed age Population
66	Update: assessment of risk for meningococcal disease associated with the Hajj 2001.	Review
67	From the Centers for Disease Control and Prevention. Serogroup W-135 meningococcal disease among travelers returning from Saudi Arabia--United States, 2000.	Review

68	Meningococcal arthritis due to group A Neisseria Mmeningitidis acquired during Mecca pilgrimage	Case Report
69	An outbreak of meningococcal infection at the time of pilgrimage in Saudi Arabia.	Mixed age Population
70	Infections due to Neisseria meningitidis serogroup a in France (August 1987 - March 1988). Relationship with mecca outbreak August 1987	Mixed age Population
71	Meningococcemia in a group of Canadian students on a European trip.	Not English

Supplementary File S3: Prevalence of meningococcal disease or carriage based on travel history

Study or Subgroup	Prevalence rate	SE	Weight	Prevalence rate IV, Random, 95% CI
**2.1.1 Mass Gathering**				
Aguilera 2002	0.03	0.01	3.9%	0.03 [0.01 , 0.05]
Alasmari A 2021	77.19	0.88	3.9%	77.19 [75.47 , 78.91]
Alborzi A 2008	4.6	0.81	3.9%	4.60 [3.01 , 6.19]
Balkhy HH 2003	0.76	0.76	3.9%	0.76 [-0.73 , 2.25]
Balkhyy HH 2004	3.2	0.95	3.9%	3.20 [1.34 , 5.06]
Bashir HE 2004	6.32	1.84	3.9%	6.32 [2.71 , 9.93]
Karima TM 2003	61.9	4.74	3.8%	61.90 [52.61 , 71.19]
Lingappa JR 2003	95.83	1.23	3.9%	95.83 [93.42 , 98.24]
Memish ZA 2017	1.11	0.42	3.9%	1.11 [0.29 , 1.93]
Metanat M 2014	1.42	0.58	3.9%	1.42 [0.28 , 2.56]
Nicolas P 2005 (Morocco)	2.67	0.27	3.9%	2.67 [2.14 , 3.20]
Nicolas P 2005 (Oman)	1.56	0.36	3.9%	1.56 [0.85 , 2.27]
Nicolas P 2005 (Sudan)	2	0.89	3.9%	2.00 [0.26 , 3.74]
Smith AW 2002	16.96	2.87	3.8%	16.96 [11.33 , 22.59]
Smith AW 2003	16.35	1.92	3.9%	16.35 [12.59 , 20.11]
Smith AW 2003 (2)	1.31	0.92	3.9%	1.31 [-0.49 , 3.11]
Smith AW 2006	1.25	0.88	3.9%	1.25 [-0.47 , 2.97]
Tezer H 2019	4.37	1.35	3.9%	4.37 [1.72 , 7.02]
Yezli S 2022	0.49	0.28	3.9%	0.49 [-0.06 , 1.04]
Yezli S 2023	0.74	0.1	3.9%	0.74 [0.54 , 0.94]
Yousuf M 2000	86.67	8.78	3.5%	86.67 [69.46 , 103.88]
**Subtotal (HKSJ[a])**			**80.7%**	**18.13 [3.83 , 32.43]**
Test for overall effect: Z = 2.66 (P = 0.008)				
Heterogeneity: Tau² (REML[b]) = 968.10; Chi² = 14372.23, df = 20 (P < 0.00001); I² = 100%				
**2.1.2 Travel**				
Jones DM 1990	7.65	2.04	3.9%	7.65 [3.65 , 11.65]
Pavli A 2016	0.11	0.05	3.9%	0.11 [0.01 , 0.21]
Tafuri S 2012	6.47	1.73	3.9%	6.47 [3.08 , 9.86]
**Subtotal (HKSJ[a])**			**11.6%**	**4.41 [-5.92 , 14.74]**
Test for overall effect: Z = 1.79 (P = 0.07)				
Heterogeneity: Tau² (REML[b]) = 16.08; Chi² = 27.14, df = 2 (P < 0.00001); I² = 90%				
**2.1.3 Both Travel and Mass Gathering**				
Dull PM 2005	11.63	2.19	3.8%	11.63 [7.34 , 15.92]
Moore PS 1988	9.45	1.25	3.9%	9.45 [7.00 , 11.90]
**Subtotal (HKSJ[a])**			**7.7%**	**9.99 [-1.94 , 21.91]**
Test for overall effect: Z = 9.20 (P < 0.00001)				
Heterogeneity: Tau² (REML[b]) = 0.00; Chi² = 0.75, df = 1 (P = 0.39); I² = 0%				
**Total (HKSJ[a])**			**100.0%**	**15.94 [4.45 , 27.43]**

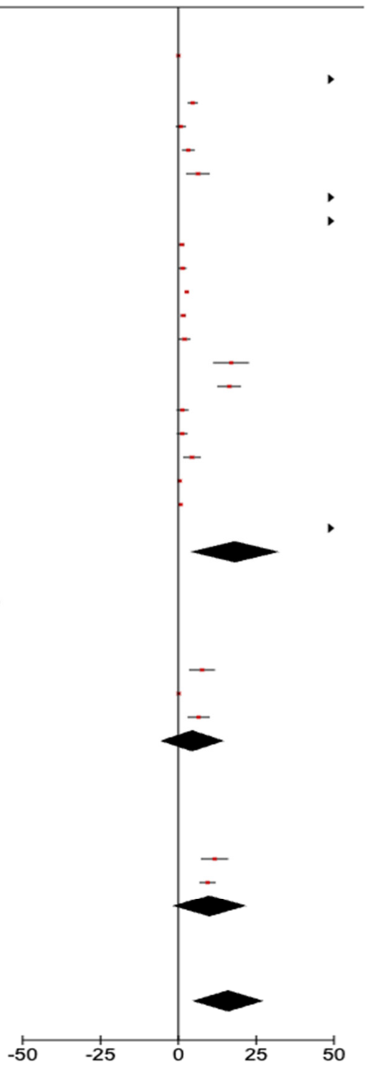

Test for overall effect: Z = 2.88 (P = 0.004)
Test for subgroup differences: Chi² = 5.99, df = 2 (P = 0.05), I² = 66.6%
Heterogeneity: Tau² (REML[b]) = 790.04; Chi² = 14484.89, df = 25 (P < 0.00001); I² = 100%

Footnotes
[a]CI calculated by Hartung-Knapp-Sidik-Jonkman method.
[b]Tau² calculated by Restricted Maximum-Likelihood method.

CI: Confidence interval; IV: Inverse variance; SE: Standard error

Supplementary File S4: Prevalence of meningococcal disease or carriage based on study design

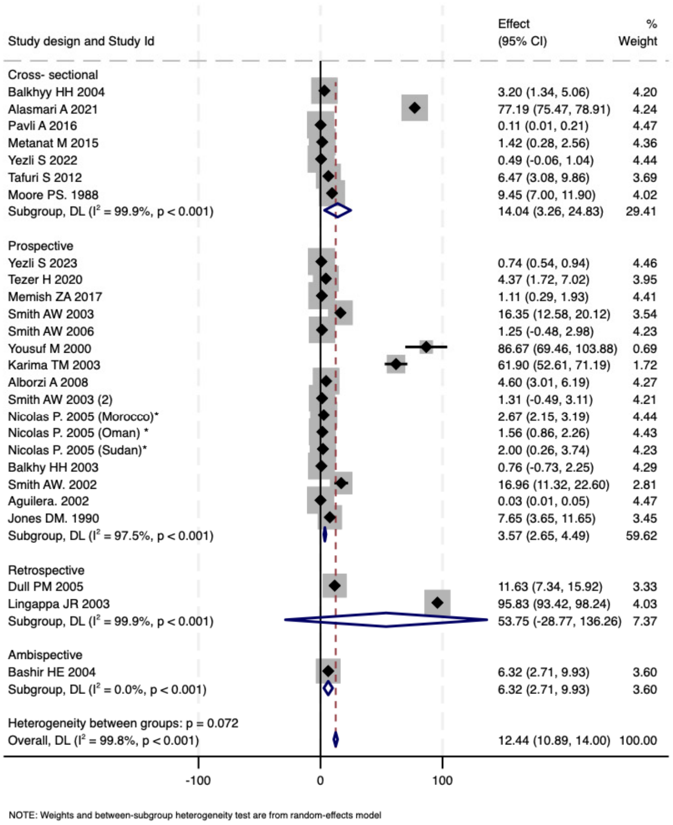

Supplementary File S5: Prevalence of meningococcal disease or carriage based on study duration/time

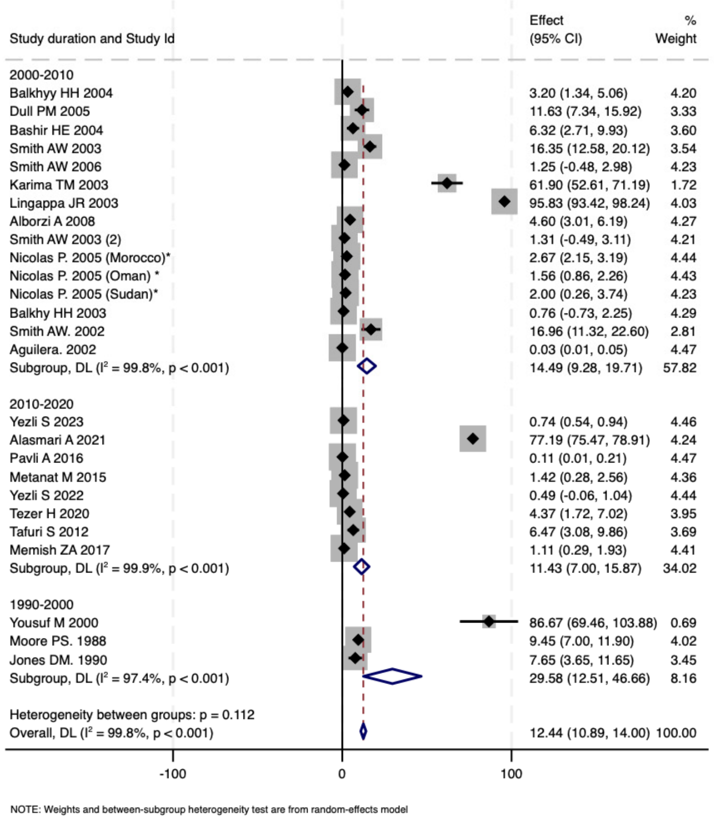

Supplementary File S6: Prevalence of meningococcal disease or carriage based on test sample

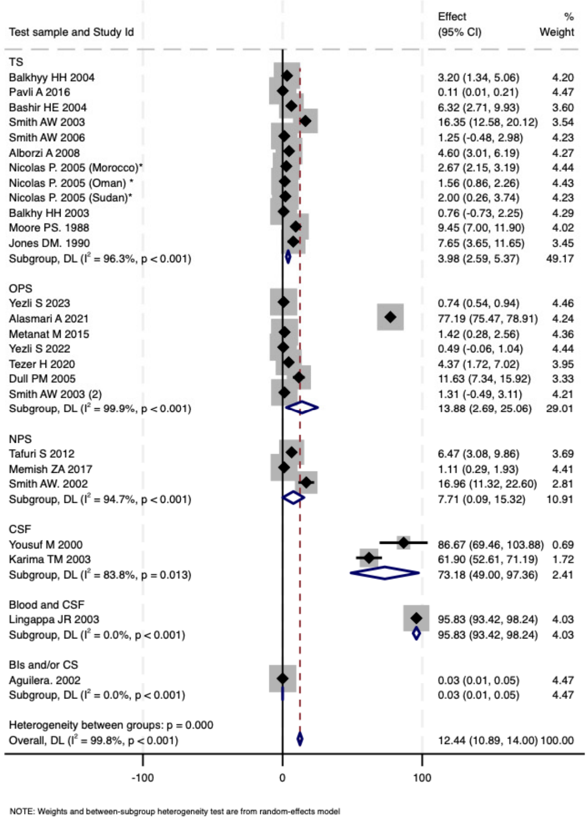

Supplementary File S7: Prevalence of serogroup A meningococcal disease or carriage based on travel history

Study or Subgroup	Prevalence rate	SE	Weight	Prevalence rate IV, Random, 95% CI
**2.3.1 Mass Gathering**				
Alasmari A 2021	0.09	0.06	11.8%	0.09 [-0.03 , 0.21]
Alborzi A 2008	0.15	0.15	11.8%	0.15 [-0.14 , 0.44]
Fazio C 2019	0.72	0.17	11.8%	0.72 [0.39 , 1.05]
Karima TM 2003	41.9	4.82	10.8%	41.90 [32.45 , 51.35]
Lingappa JR 2003	22.73	2.58	11.5%	22.73 [17.67 , 27.79]
Moore PS 1988	6.55	1.05	11.7%	6.55 [4.49 , 8.61]
Yezli S 2022	0.32	0.23	11.8%	0.32 [-0.13 , 0.77]
Yousuf M 2000	46.67	12.88	7.3%	46.67 [21.43 , 71.91]
**Subtotal (HKSJ[a])**			**88.3%**	**13.11 [-2.43 , 28.65]**
Test for overall effect: Z = 2.09 (P = 0.04)				
Heterogeneity: Tau² (REML[b]) = 298.16; Chi² = 213.79, df = 7 (P < 0.00001); I² = 100%				
**2.3.2 Travel**				
Jones DM 1990	0.59	0.59	11.7%	0.59 [-0.57 , 1.75]
**Subtotal**			**11.7%**	**0.59 [-0.57 , 1.75]**
Test for overall effect: Z = 1.00 (P = 0.32)				
Heterogeneity: Not applicable				
**Total (HKSJ[a])**			**100.0%**	**11.51 [-2.13 , 25.15]**

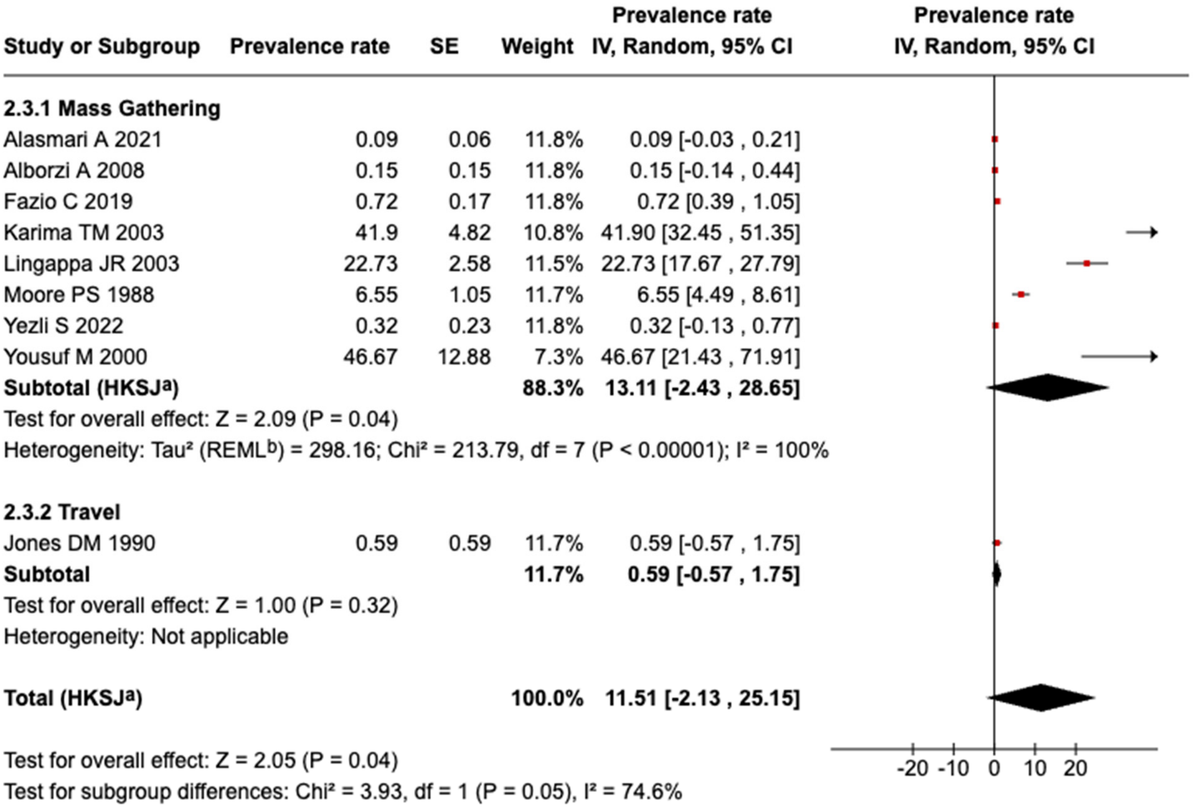

Test for overall effect: Z = 2.05 (P = 0.04)
Test for subgroup differences: Chi² = 3.93, df = 1 (P = 0.05), I² = 74.6%
Heterogeneity: Tau² (REML[b]) = 267.19; Chi² = 214.23, df = 8 (P < 0.00001); I² = 100%

Footnotes
[a]CI calculated by Hartung-Knapp-Sidik-Jonkman method.
[b]Tau² calculated by Restricted Maximum-Likelihood method.

CI: Confidence interval; IV: Inverse variance; SE: Standard error

Supplementary File S8: Prevalence of serogroup B meningococcal disease or carriage based on travel history.

Study or Subgroup	Prevalence rate	SE	Weight	Prevalence rate IV, Random, 95% CI
**2.4.1 Mass Gathering**				
Alasmari A 2021	0.44	0.14	7.8%	0.44 [0.17 , 0.71]
Alborzi A 2008	0.3	0.21	7.8%	0.30 [-0.11 , 0.71]
Balkhyy HH 2004	0.29	0.29	7.8%	0.29 [-0.28 , 0.86]
Bashir HE 2004	1.72	0.99	7.8%	1.72 [-0.22 , 3.66]
Fazio C 2019	52.99	1.03	7.8%	52.99 [50.97 , 55.01]
Karima TM 2003	0.95	0.95	7.8%	0.95 [-0.91 , 2.81]
Nicolas P 2005 (Morocco)	0.34	0.1	7.8%	0.34 [0.14 , 0.54]
Nicolas P 2005 (Oman)	0.35	0.17	7.8%	0.35 [0.02 , 0.68]
Tezer H 2019	0.44	0.44	7.8%	0.44 [-0.42 , 1.30]
Yezli S 2022	0.16	0.16	7.8%	0.16 [-0.15 , 0.47]
Yezli S 2023	0.03	0.02	7.8%	0.03 [-0.01 , 0.07]
Yousuf M 2000	6.67	6.44	6.5%	6.67 [-5.95 , 19.29]
**Subtotal (HKSJ[a])**			**92.2%**	**5.36 [-4.28 , 15.00]**
Test for overall effect: Z = 1.22 (P = 0.22)				
Heterogeneity: Tau² (REML[b]) = 229.32; Chi² = 2668.07, df = 11 (P < 0.00001); I² = 100%				
**2.4.2 Both Travel and Mass Gathering**				
Dull PM 2005	2.33	1.03	7.8%	2.33 [0.31 , 4.35]
**Subtotal**			**7.8%**	**2.33 [0.31 , 4.35]**
Test for overall effect: Z = 2.26 (P = 0.02)				
Heterogeneity: Not applicable				
**Total (HKSJ[a])**			**100.0%**	**5.12 [-3.67 , 13.91]**

Test for overall effect: Z = 1.26 (P = 0.21)
Test for subgroup differences: Chi² = 0.45, df = 1 (P = 0.50), I² = 0%
Heterogeneity: Tau² (REML[b]) = 210.80; Chi² = 2672.85, df = 12 (P < 0.00001); I² = 100%

Prevalence rate IV, Random, 95% CI

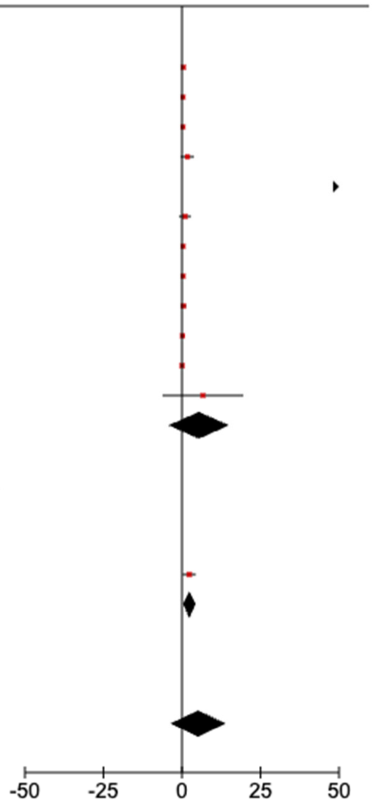

Footnotes
[a]CI calculated by Hartung-Knapp-Sidik-Jonkman method.
[b]Tau² calculated by Restricted Maximum-Likelihood method.

CI: Confidence interval; IV: Inverse variance; SE: Standard error

Supplementary File S9: Prevalence of serogroup W135 meningococcal disease or carriage based on travel history

Study or Subgroup	Prevalence rate	SE	Weight	Prevalence rate IV, Random, 95% CI
**2.2.1 Mass Gathering**				
Alborzi A 2008	0.15	0.15	7.1%	0.15 [-0.14 , 0.44]
Balkhy HH 2003	0.76	0.76	7.0%	0.76 [-0.73 , 2.25]
Balkhyy HH 2004	0.58	0.41	7.1%	0.58 [-0.22 , 1.38]
Bashir HE 2004	0.57	0.57	7.0%	0.57 [-0.55 , 1.69]
Karima TM 2003	18.1	3.76	6.1%	18.10 [10.73 , 25.47]
Lingappa JR 2003	35.23	2.94	6.5%	35.23 [29.47 , 40.99]
Nicolas P 2005 (Morocco)	0.87	0.16	7.1%	0.87 [0.56 , 1.18]
Nicolas P 2005 (Oman)	0.78	0.26	7.1%	0.78 [0.27 , 1.29]
Nicolas P 2005 (Sudan)	2	0.89	7.0%	2.00 [0.26 , 3.74]
Smith AW 2002	15.2	2.75	6.5%	15.20 [9.81 , 20.59]
Smith AW 2003	15.01	1.85	6.8%	15.01 [11.38 , 18.64]
Smith AW 2003 (2)	1.31	0.92	7.0%	1.31 [-0.49 , 3.11]
Yousuf M 2000	13.33	8.78	3.8%	13.33 [-3.88 , 30.54]
**Subtotal (HKSJ[a])**			**86.1%**	**7.51 [1.05 , 13.96]**
Test for overall effect: Z = 2.54 (P = 0.01)				
Heterogeneity: Tau^2 (REML[b]) = 107.28; Chi^2 = 266.32, df = 12 (P < 0.00001); I^2 = 100%				
**2.2.2 Travel**				
Tafuri S 2012	1.99	0.99	7.0%	1.99 [0.05 , 3.93]
**Subtotal**			**7.0%**	**1.99 [0.05 , 3.93]**
Test for overall effect: Z = 2.01 (P = 0.04)				
Heterogeneity: Not applicable				
**2.2.3 Both Travel and Mass Gathering**				
Dull PM 2005	4.65	1.44	6.9%	4.65 [1.83 , 7.47]
**Subtotal**			**6.9%**	**4.65 [1.83 , 7.47]**
Test for overall effect: Z = 3.23 (P = 0.001)				
Heterogeneity: Not applicable				
**Total (HKSJ[a])**			**100.0%**	**6.87 [1.35 , 12.39]**

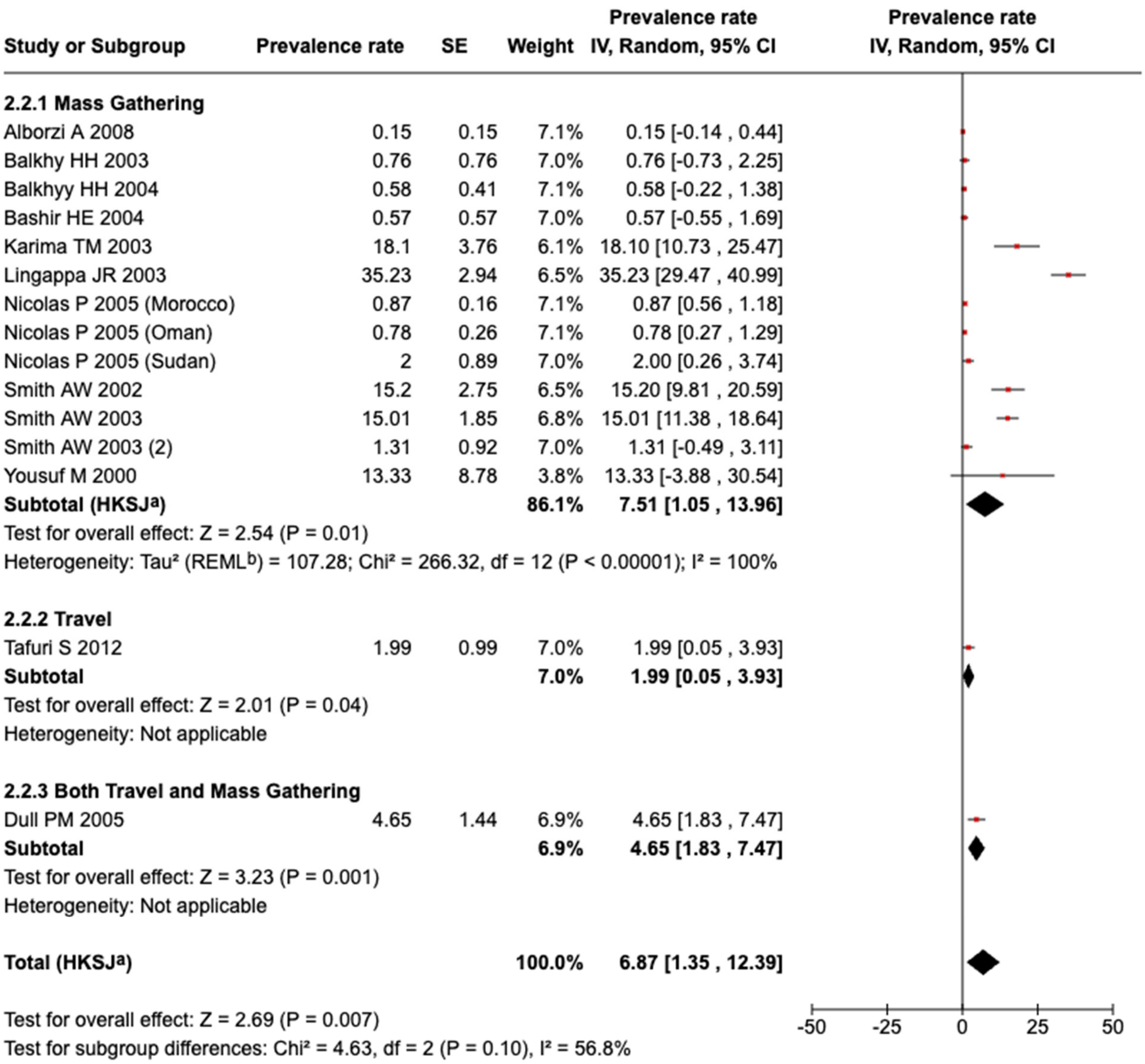

Test for overall effect: Z = 2.69 (P = 0.007)
Test for subgroup differences: Chi^2 = 4.63, df = 2 (P = 0.10), I^2 = 56.8%
Heterogeneity: Tau^2 (REML[b]) = 92.15; Chi^2 = 275.67, df = 14 (P < 0.00001); I^2 = 100%

Footnotes
[a]CI calculated by Hartung-Knapp-Sidik-Jonkman method.
[b]Tau^2 calculated by Restricted Maximum-Likelihood method.

CI: Confidence interval; IV: Inverse variance; SE: Standard error

Supplementary File S10: Prevalence of Non-groupable serogroup meningococcal disease or carriage based on travel history

Study or Subgroup	Prevalence rate	SE	Weight	Prevalence rate IV, Random, 95% CI
**2.7.1 Mass Gathering**				
Alborzi A 2008	3.12	0.67	9.5%	3.12 [1.81 , 4.43]
Balkhy HH 2003	3.82	1.67	5.4%	3.82 [0.55 , 7.09]
Balkhyy HH 2004	2.33	0.81	8.9%	2.33 [0.74 , 3.92]
Bashir HE 2004	3.45	1.38	6.4%	3.45 [0.75 , 6.15]
Jones DM 1990	6.47	1.89	4.7%	6.47 [2.77 , 10.17]
Karima TM 2003	0.95	0.95	8.2%	0.95 [-0.91 , 2.81]
Nicolas P 2005 (Morocco)	1.32	0.19	10.9%	1.32 [0.95 , 1.69]
Nicolas P 2005 (Oman)	0.43	0.19	10.9%	0.43 [0.06 , 0.80]
Smith AW 2006	0.63	0.62	9.7%	0.63 [-0.59 , 1.85]
Yezli S 2023	0.03	0.02	11.1%	0.03 [-0.01 , 0.07]
**Subtotal (HKSJ[a])**			**85.6%**	**1.71 [0.51 , 2.92]**
Test for overall effect: Z = 3.51 (P = 0.0004)				
Heterogeneity: Tau² (REML[b]) = 1.68; Chi² = 103.00, df = 9 (P < 0.00001); I² = 96%				
**2.7.2 Both Travel and Mass Gathering**				
Dull PM 2005	6.98	1.74	5.1%	6.98 [3.57 , 10.39]
Moore PS 1988	2.91	0.72	9.2%	2.91 [1.50 , 4.32]
**Subtotal (HKSJ[a])**			**14.4%**	**4.64 [-20.92 , 30.20]**
Test for overall effect: Z = 2.31 (P = 0.02)				
Heterogeneity: Tau² (REML[b]) = 6.51; Chi² = 4.67, df = 1 (P = 0.03); I² = 79%				
**Total (HKSJ[a])**			**100.0%**	**2.19 [0.91 , 3.47]**

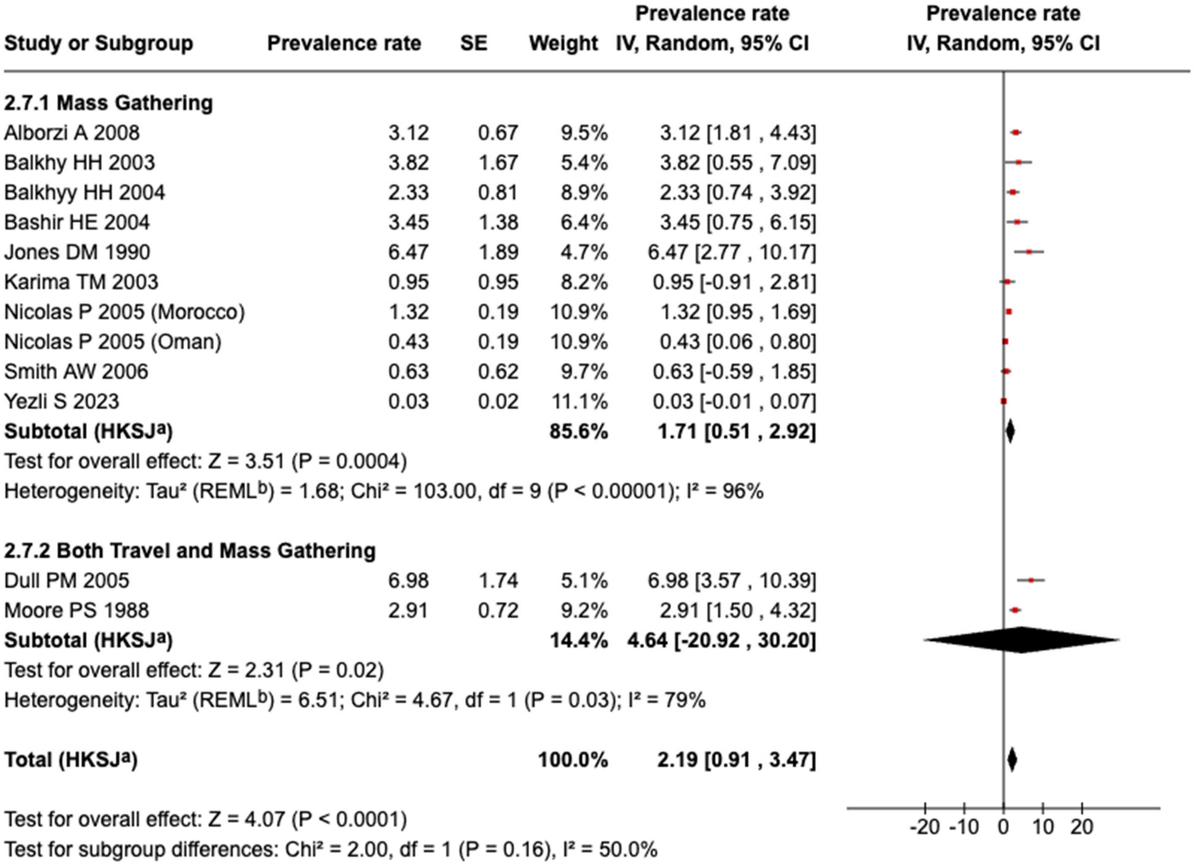

Test for overall effect: Z = 4.07 (P < 0.0001)
Test for subgroup differences: Chi² = 2.00, df = 1 (P = 0.16), I² = 50.0%
Heterogeneity: Tau² (REML[b]) = 2.61; Chi² = 134.54, df = 11 (P < 0.00001); I² = 97%

Footnotes
[a]CI calculated by Hartung-Knapp-Sidik-Jonkman method.
[b]Tau² calculated by Restricted Maximum-Likelihood method.

CI: Confidence interval; IV: Inverse variance; SE: Standard error

Supplementary File S11: Risk of meningococcal disease or carriage based on age in years

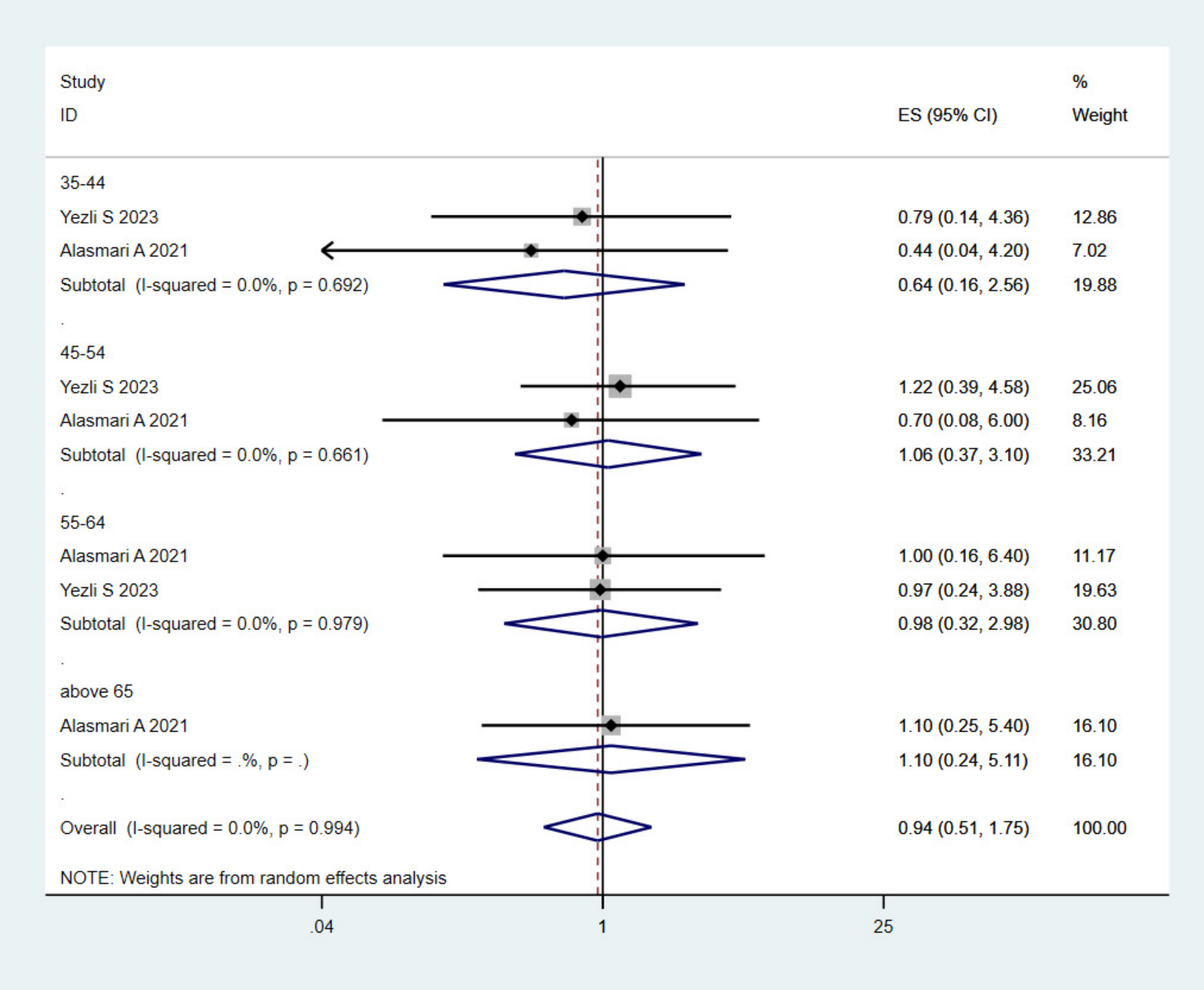

CI: Confidence interval; Effect size (ES) used in this analysis was Risk Ratio

Supplementary File S12: Risk of meningococcal disease or carriage for male gender

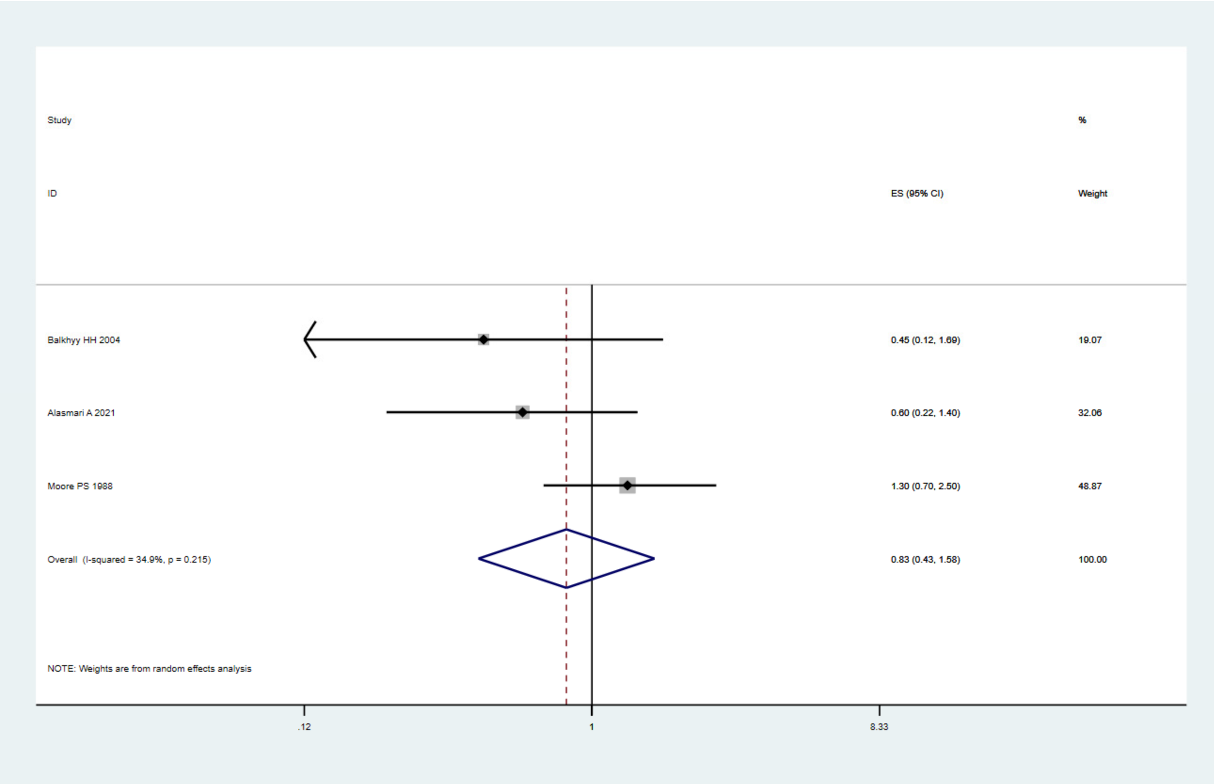

CI: Confidence interval; Effect size (ES) used in this analysis was Risk Ratio

Supplementary File S13: Risk of meningococcal disease or carriage among those with any smoking history

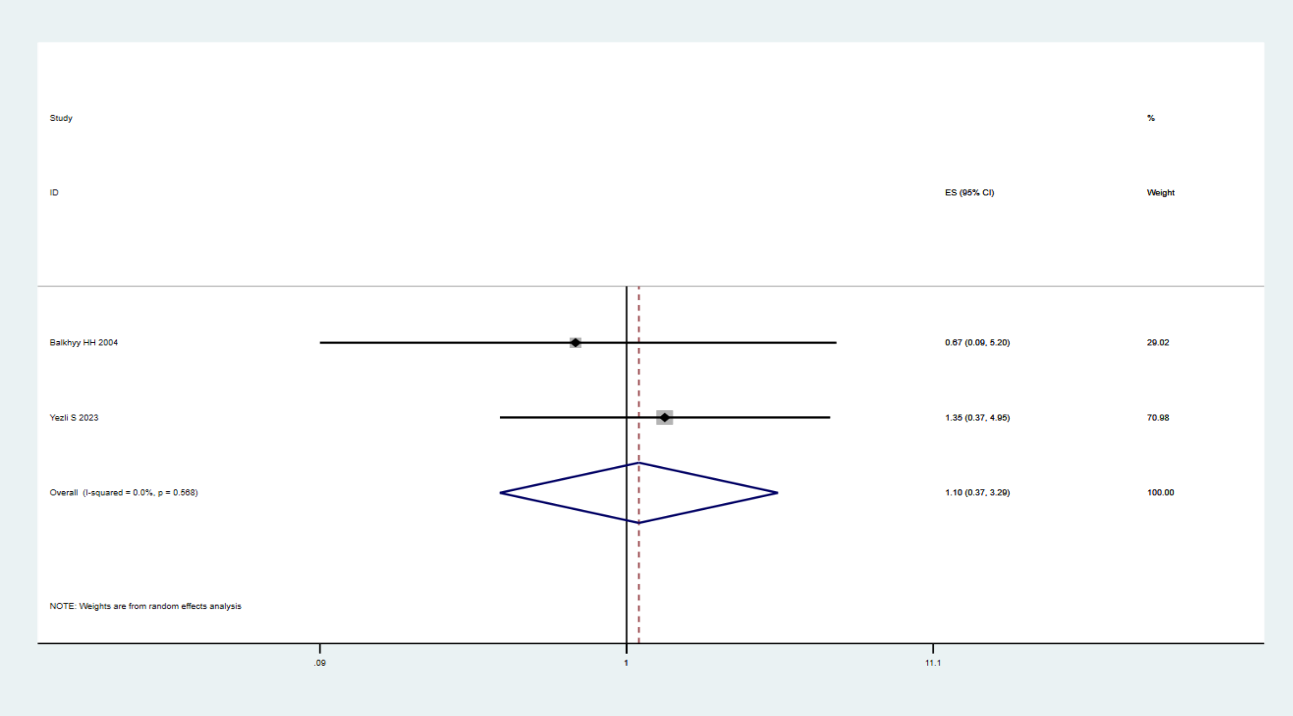

CI: Confidence interval; Effect size (ES) used in this analysis was Risk Ratio

Supplementary File S14: Risk of meningococcal disease or carriage among those with no vaccination

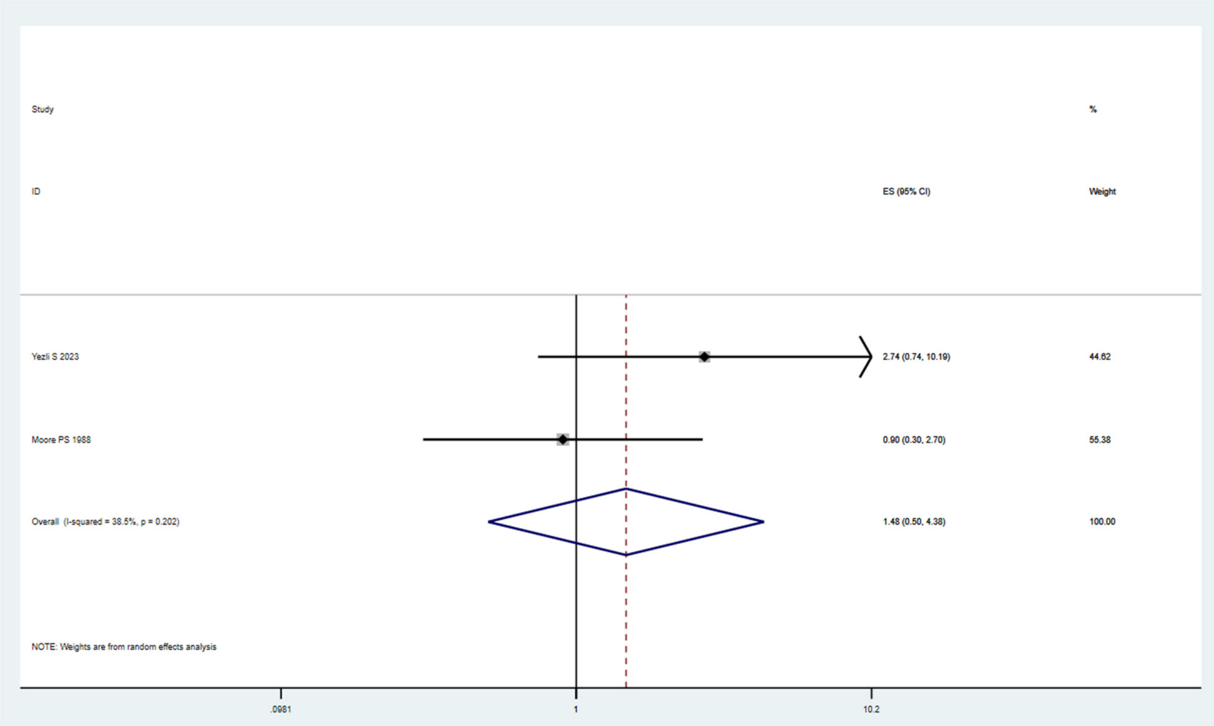

CI: Confidence interval; Effect size (ES) used in this analysis was Risk Ratio

Supplementary File S15: Funnel plot for meningococcal disease or carriage

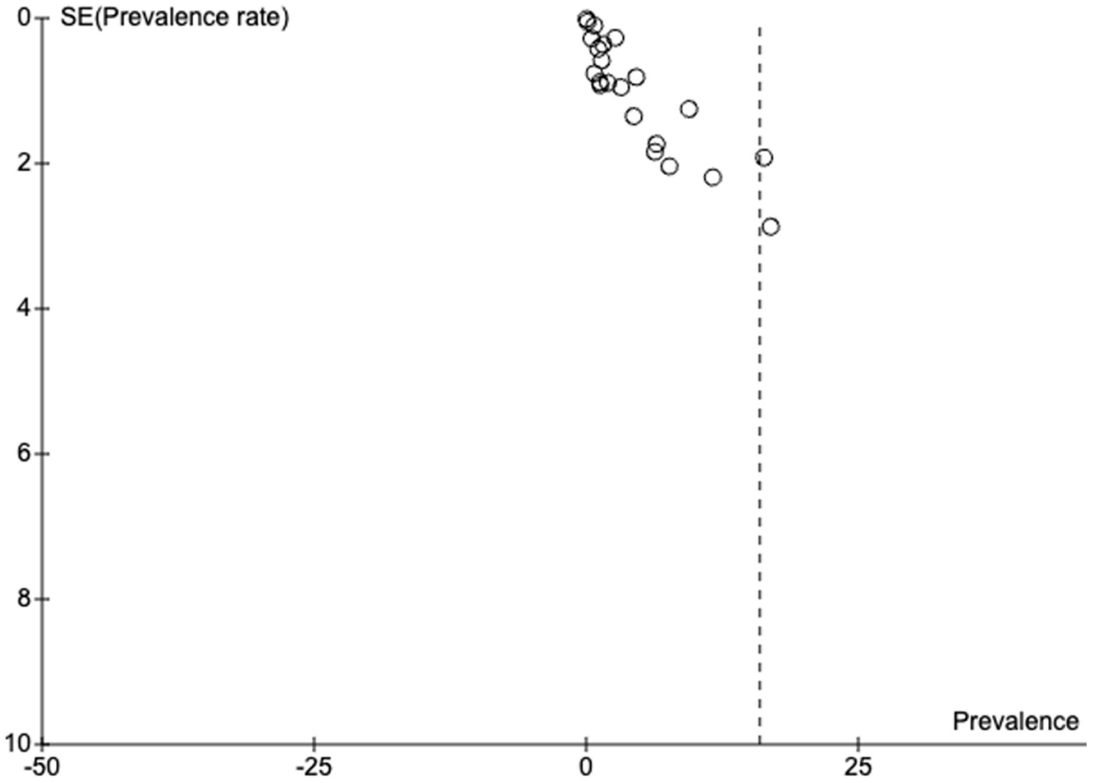

X axis represent the prevalence rate of meningococcal disease and the Y axis indicates the standard error; Each circle represents individual studies in the analysis

Supplementary File S16: Funnel plot for group B meningococcal disease or carriage

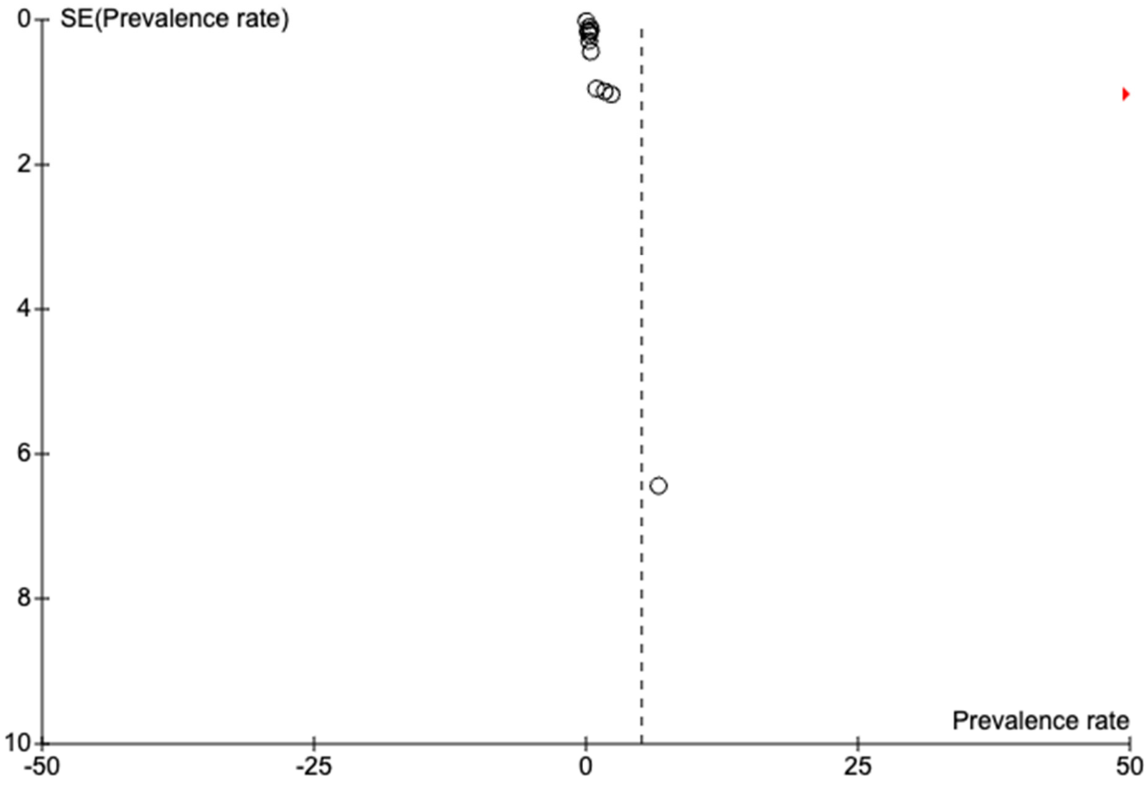

X axis represent the prevalence rate of meningococcal B serotype; and Y axis indicate the standard error; Each circle represents individual studies in the analysis

Supplementary File S17: Funnel plot for group W135 meningococcal disease or carriage

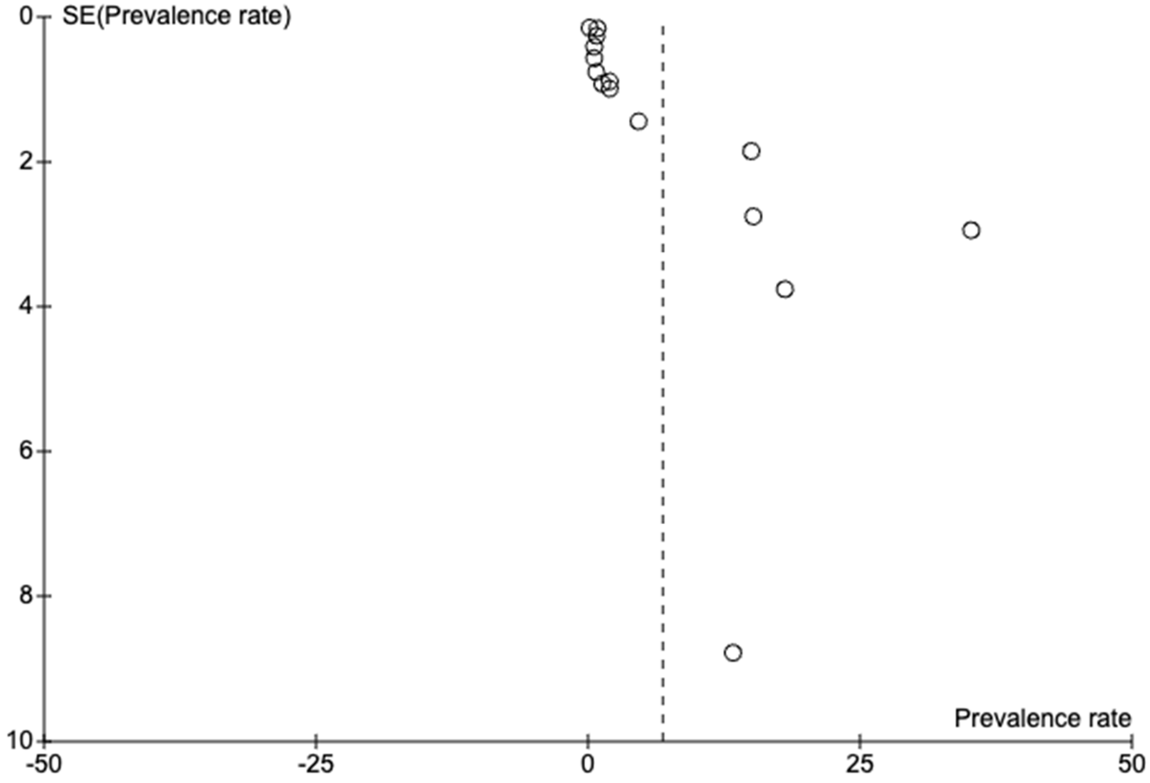

X axis represent the prevalence rate of meningococcal W-135 serotype; and Y axis indicate the standard error; Each circle represents individual studies in the analysis

Supplementary File S18: Funnel plot for group W135 meningococcal disease or carriage

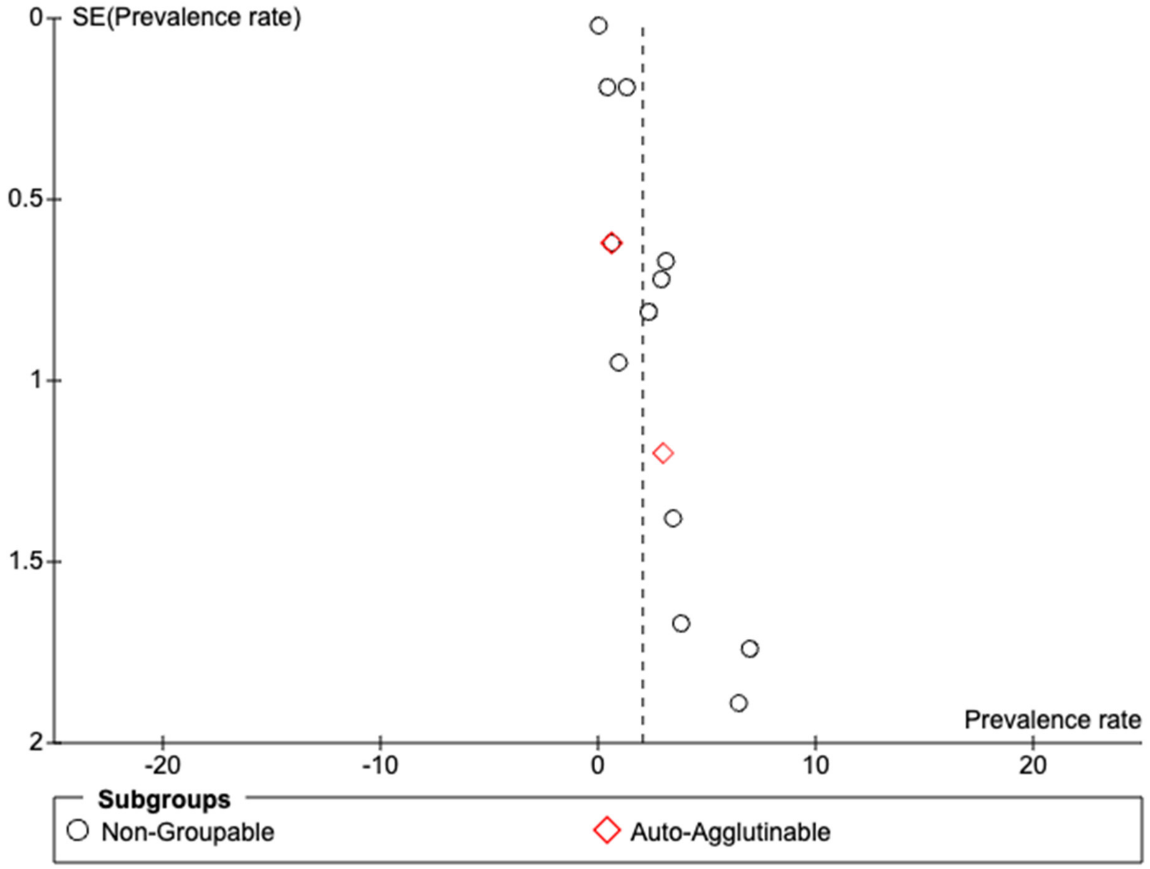

X axis represent the prevalence rate of non-groupable and auto-agglutinable serotypes; and Y axis indicate the standard error; Each circle represent individual studies in the analysis of non-groupable serotypes. The triangles represent studies reporting auto-agglutinable serotypes.

Supplementary File S19: Sensitivity analysis

Study or Subgroup	Prevalence rate	SE	Weight	Prevalence rate IV, Random, 95% CI
Aguilera 2002	0.03	0.01	4.2%	0.03 [0.01 , 0.05]
Alasmari A 2021	77.19	0.88	4.2%	77.19 [75.47 , 78.91]
Alborzi A 2008	4.6	0.81	4.2%	4.60 [3.01 , 6.19]
Balkhyy HH 2004	3.2	0.95	4.2%	3.20 [1.34 , 5.06]
Bashir HE 2004	6.32	1.84	4.2%	6.32 [2.71 , 9.93]
Dull PM 2005	11.63	2.19	4.2%	11.63 [7.34 , 15.92]
Jones DM 1990	7.65	2.04	4.2%	7.65 [3.65 , 11.65]
Karima TM 2003	61.9	4.74	4.0%	61.90 [52.61 , 71.19]
Lingappa JR 2003	95.83	1.23	4.2%	95.83 [93.42 , 98.24]
Memish ZA 2017	1.11	0.42	4.2%	1.11 [0.29 , 1.93]
Metanat M 2014	1.42	0.58	4.2%	1.42 [0.28 , 2.56]
Moore PS 1988	9.45	1.25	4.2%	9.45 [7.00 , 11.90]
Nicolas P 2005 (Morocco)	2.67	0.27	4.2%	2.67 [2.14 , 3.20]
Nicolas P 2005 (Oman)	1.56	0.36	4.2%	1.56 [0.85 , 2.27]
Nicolas P 2005 (Sudan)	2	0.89	4.2%	2.00 [0.26 , 3.74]
Pavli A 2016	0.11	0.05	4.2%	0.11 [0.01 , 0.21]
Smith AW 2002	16.96	2.87	4.1%	16.96 [11.33 , 22.59]
Smith AW 2003	16.35	1.92	4.2%	16.35 [12.59 , 20.11]
Smith AW 2003 (2)	1.31	0.92	4.2%	1.31 [-0.49 , 3.11]
Smith AW 2006	1.25	0.88	4.2%	1.25 [-0.47 , 2.97]
Tafuri S 2012	6.47	1.73	4.2%	6.47 [3.08 , 9.86]
Tezer H 2019	4.37	1.35	4.2%	4.37 [1.72 , 7.02]
Yezli S 2022	0.49	0.28	4.2%	0.49 [-0.06 , 1.04]
Yezli S 2023	0.74	0.1	4.2%	0.74 [0.54 , 0.94]
**Total (HKSJ[a])**			**100.0%**	**13.87 [3.00 , 24.75]**

Prevalence rate IV, Random, 95% CI

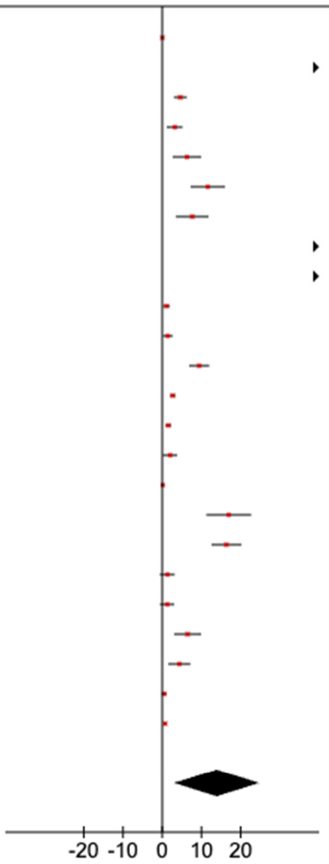

Test for overall effect: Z = 2.64 (P = 0.008)
Test for subgroup differences: Not applicable
Heterogeneity: Tau² (REML[b]) = 659.22; Chi² = 14386.76, df = 23 (P < 0.00001); I² = 100%

Footnotes
[a]CI calculated by Hartung-Knapp-Sidik-Jonkman method.
[b]Tau² calculated by Restricted Maximum-Likelihood method.

CI: Confidence interval; Effect size (ES) used in this analysis was Risk Ratio; IV: Inverse variance
